# Practical Methodology in Kinetic Modeling for Complex Reactions: Weighted Error Manipulation to Allow Effective Model Evaluation in a Borderline S_N_ Reaction

**DOI:** 10.1021/acsomega.4c09609

**Published:** 2025-02-24

**Authors:** Yuya Orito

**Affiliations:** Process Technology Research Laboratories, Technology Development Supervisory Department, Technology Division, Daiichi Sankyo Co., Ltd., Hiratsuka, Kanagawa 254-0014, Japan

## Abstract

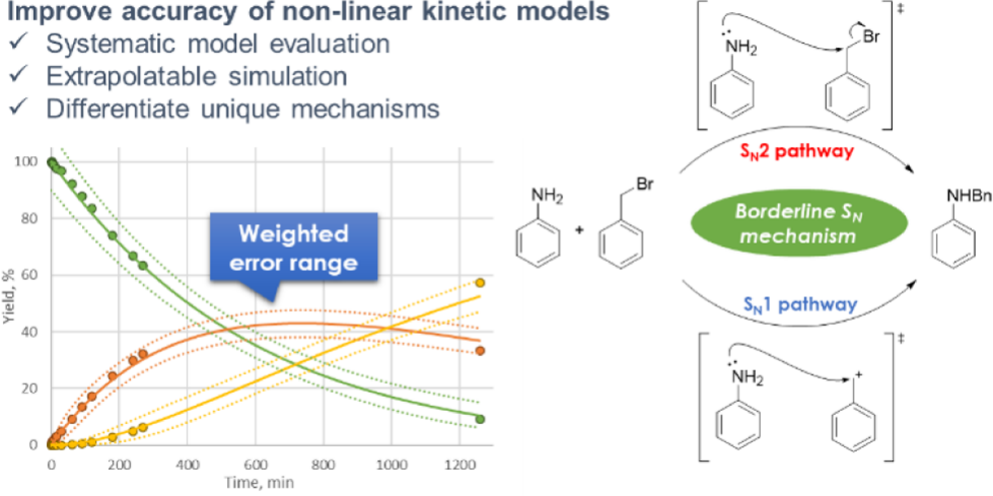

This paper details a novel and mechanism-oriented approach to kinetic modeling of complex chemical reactions, which focuses on the importance of a detailed understanding of the reaction mechanism and appropriate experimental data collection in the development and evaluation of accurate reaction models. Instead of using traditional statistical indices centered on the experimental data, this approach introduces a fitting index based on a weighted continuous error range centered on simulated data to accomplish effective model evaluation. Also, as a practical example, the method was applied to distinguish a borderline S_N_ reaction mechanism involving five elementary steps, and the results showed improved model fit compared to models involving solely S_N_1 or S_N_2 mechanisms. This approach provides a new aspect for model evaluation and validation in kinetic modeling based on both mechanistic understanding and experimental data.

## Introduction

Kinetic modeling of chemical reactions is a powerful technique for reaction analysis and control strategy and has recently attracted significant research attention.^1−12^ The most useful feature of a reaction kinetic model should be extrapolability, i.e., the capability to predict reactions under unknown conditions outside of the input data range used for modeling, due to the nature of the rate law as a physical model.^13^ Thus, the application field of kinetic models is not limited to utilizing self-reproducibility (prediction *inside* the input data range) but is also recognized as a versatile tool for predictive reaction design and process development. However, there are challenges when trying to fit models in practice for complex chemical reactions, which consist of multiple elementary steps, even when utilizing high-function modeling and simulation software tools and modern experimental approaches. In many cases, the “best-fitted” model obtained through statistical regressions would not produce prediction curves satisfactorily similar to experimental results in extrapolation, possibly due to over-approximation of the complex reaction kinetics. This discrepancy should be attributed to the fact that the kinetic model of a reaction refers to the rate law of each reaction, i.e., a set of simultaneous rate equations, involving complex, nonlinear rate-determining step (rds) such as competing, consecutive reactions, pre/post-equilibria, etc., which are usually quite difficult to resolve into the correct set of elementary steps.^14^ Kinetic models with fractional orders are well investigated since they can produce satisfactory results when used for interpolative (inside or close conditions to modeling experiments) simulation. Meanwhile, these fractional orders may lead to prediction failure in extrapolation because the rate law must have an integer order for all elements of the reaction (substances, catalysts, etc.) to avoid over-approximation. Hence, to achieve extrapolative reaction prediction, a systematic approach with a reasonable understanding of the mechanism based on both chemical knowledge and experimental facts is required to improve the quality of a reaction model.

Herein, the key considerations in the numerical evaluation and comparison of kinetic models are discussed by focusing on some specific issues in the experiment and analysis of chemical reactions, subject to reproducing experimental data in extrapolation. A practical example of its application in discriminating a borderline S_N_ reaction mechanism is demonstrated.

## Results and Discussion

### General Points to Consider in Reaction Model Building and Selection

In contrast to simple first or second-order reactions, in which the rds mainly consists of a single elementary step, the reaction time course plot in more multistep reactions results in complex nonlinear curves. To estimate model parameters such as activation energy and pre-exponential factor, nonlinear least-squares regression is a well-established and commonly employed method in the modeling process.^15−17^ However, the main challenge in kinetic model simulation is to select an appropriate model, i.e., a set of elementary steps connected to the rds. It is important to strike a balance, avoiding over-approximation by including too few steps, while also preventing excessive computational resource usage and overfitting by including too many steps. In this perspective, visual reaction analysis approaches such as RPKA and VTNA are widely used as effective data implementation methods for reaction analysis.^1−4^ By using these methodologies, inconsistency in rate law (due to catalyst/substance decomposition, etc.) and the order of each substance can be detected from reaction time course data to help effective kinetic modeling. However, it is still difficult to obtain a model capable of extrapolation, since one must discriminate the correct model from multiple possibilities, identifying hidden elementary steps that may consist of undetectable transient intermediates, instability, or analytical limitations. Unfortunately, the “least-squares” approach only affords the best fit between the “given” set of equations and data points, and statistical indicators such as confidence intervals are not capable of distinguishing whether the model itself is chosen appropriately. Moreover, adding even a single elementary step to an existing reaction model technically adds at least two more degrees of freedom, which may result in much wider confidence intervals leading to convergence problems. Thus, it is important to be cautious when introducing “imaginary” elementary steps unless there is experimental evidence that supports their actual existence or any other reason to justify it logically based on chemical knowledge. On the other hand, collecting an appropriate experimental data set is also of importance since it is the only available direct information on the target reaction; in other words, it reflects the reaction mechanism even though multiple types of error may be involved. Consequently, an extensive understanding of the reaction and investigation of rate-determining elementary steps based on the acquisition of real experimental data are inevitably required to develop an appropriate reaction model to improve the accuracy of simulation.

### Error Managing Strategy in the Modeling Process

**Figure 1:**
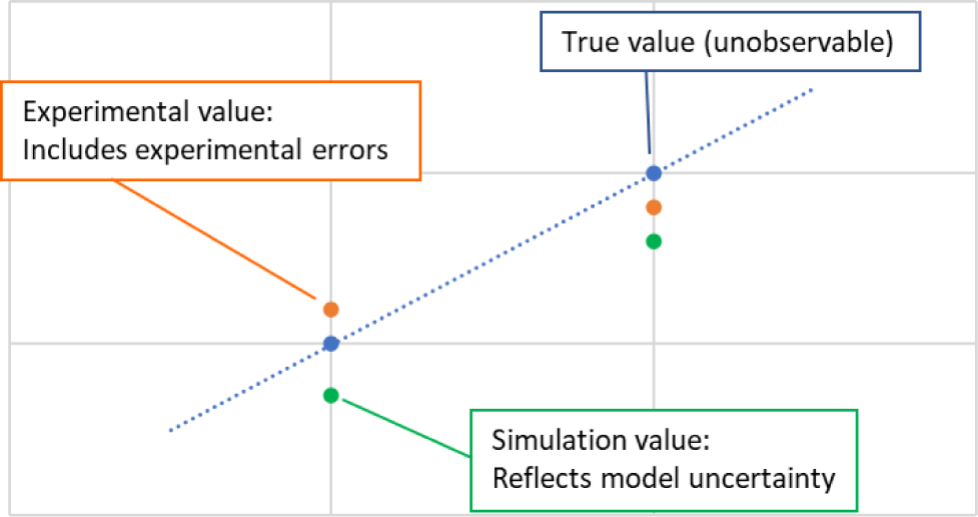
Schematic illustration of the differences between true values and experimental/simulated values.

Despite the issues stated above, nonlinear regression remains an invaluable method for parameter fitting, provided that the model can be chosen appropriately. However, it should be noted that this statistical approach assumes the condition of ideal data in which the selected input model exactly matches the true rate law and outputs true values, with all experimental errors following a normal distribution. In contrast, during the process of kinetic modeling and simulation of actual chemical reactions, there exist multiple errors to consider arising from various factors. When model fitting is conducted using experimental data, two distinct types of errors must be considered: experimental error and model error. Importantly, the observable “error” actually refers to the discrepancy between the experimental data points and the simulation results obtained by using a hypothetical kinetic model, with both having deviations from the true value, which is purely theoretical and not directly measurable (Figure 1). Thus, the distance between prediction and experimental data is the primary parameter to minimize to improve model quality.

Regarding experimental error, when collecting experimental data, there is inevitably some degree of scatter related to every aspect of conducting the experiment, such as stoichiometry, temperature, way of addition, mixing, and other inconsistencies or faults in manipulation, along with sampling errors in terms of time accuracy, quenching method, quenching temperature, analytical instruments setup, etc.^1^ Moreover, not all of these errors are ideally random; rather, there are many biases, such as systematic errors of analytical conditions, sampling delays in fast reactions, exothermic quenching, NMR acquisition time, etc., which make the observable error less uniform and thus deviate from a normal distribution. This makes curve regression difficult, although these biases tend to be identifiable and correctable by thorough investigation of experimental conditions. Further, experimental (quantitative) data in lower yields fundamentally have a larger relative error, which arises from data noise in the analysis, whereas the impact is smaller at higher yields. Also, early-stage data are sensitive to sampling timing since the reaction rate is fast, while late-stage data are affected less as the rate of concentration change is relatively smaller.

On the other hand, the proposed kinetic model itself will also have errors, in other words, uncertainty. Even with a deep understanding of the reaction mechanism, there will inevitably be minor approximations of the real reaction. It is nearly impossible (and actually not practical) to include all existing single elementary steps, especially those with very low contributions to observable kinetics (or rds), in the kinetic model as multiple simultaneous rate equations. Thus, the sum of these undetectable minor reactions will lead to simulation errors.

Importantly, since the reaction kinetics are a physical model, the simulation is expected to have the same accuracy even outside of the input data range; in other words, the prediction is extrapolatable, provided that the model and the reaction are mechanistically consistent. Thus, the extrapolability can be a good indicator of the model’s validity and is easily tested by a double-check of simulation results with one or more additional experimental measurements. From this point of view, one needs to evaluate how the simulated curve can reproduce (overlay) the experimental data to investigate the model’s quality, in consideration of both experimental errors and the consistency with a mechanistic understanding.

### Collecting Desirable Experimental Data for Kinetic Modeling

**Figure 2:**
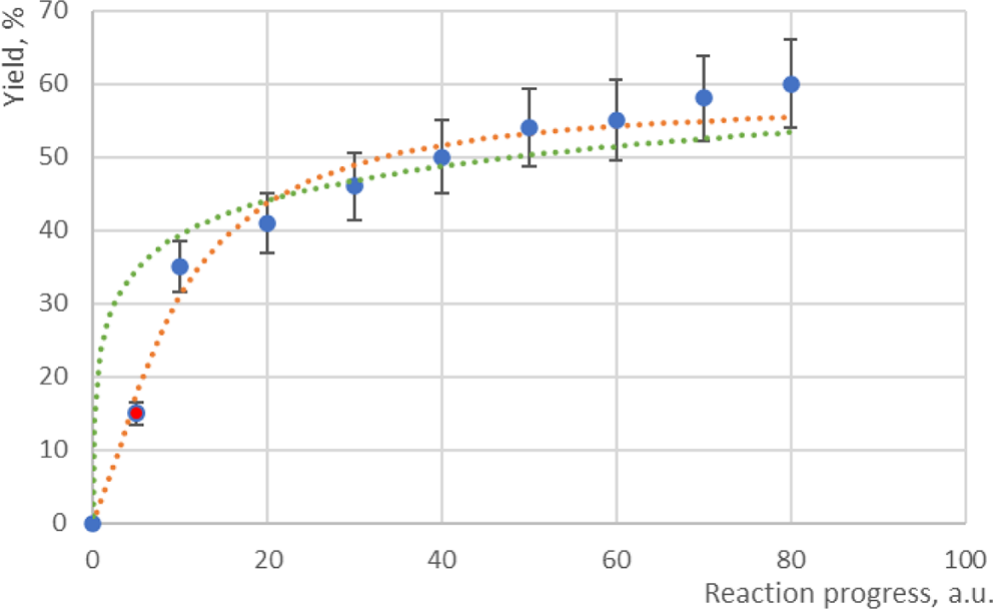
Example of the impact of early data points on curve-shape determination. Adding the early data point (red circle) into data series with equal intervals (blue circle) can distinguish the curve shape between exponential (green) and sigmoidal (orange), while frequent sampling in later stage plays less importance in regression.

To achieve appropriate error manipulation, the form of the experimental data set is of importance. Recently, real-time reaction monitoring techniques, known as Process Analytical Technology (PAT),^1,18−20^ have been actively developed to obtain continuous data from chemical reactions. These real-time reaction trends are especially effective in detecting deviations from steady state or anomalies in reaction behavior. However, they are rather weak to systematic (bias) errors that can cause parallel shifts of the curves, resulting in fitting failure even if the reaction model is chosen appropriately. This is mainly due to the nonuniformity of the contribution of each data point in the model fitting process. Generally, kinetic model simulations produce a shape of curve that is dependent on the rate law, such as exponential, sigmoidal, or formal higher (>2nd) order kinetics, etc., so experimental data points for modeling purposes need to be capable of distinguishing the curve shape. As discussed in the previous section, the data points have a relatively large rate of concentration (yield) change at the beginning of the reaction and greatly influence the shape of the curve; therefore, frequent data collection is required in modeling experiments, as shown in Figure 2. On the other hand, in the later stages of the reaction, the rate of concentration change becomes slow and gradual, so longer data intervals are acceptable as their influence on the curve shape is expected to be minor.

Hence, if all data points are evaluated evenly throughout the reaction time-course, it may risk convergence failure or overfitting. Furthermore, evaluating too many data points, such as continuous data, may lead to the accumulation of a bias error or underestimation of the error level by late-stage data, while both can be managed together with random errors when the number of data points is decreased. Consequently, exponential and sparse interval sampling (i.e., 1, 2, 4, 8,... min, for example) is thought to be preferable for modeling experiments. Also, as the rate constant is affected by the reaction temperature, the actual internal reaction temperature should be monitored along with concentration data.

### Numerical Evaluation of Quality of a Kinetic Model

**Figure 3:**
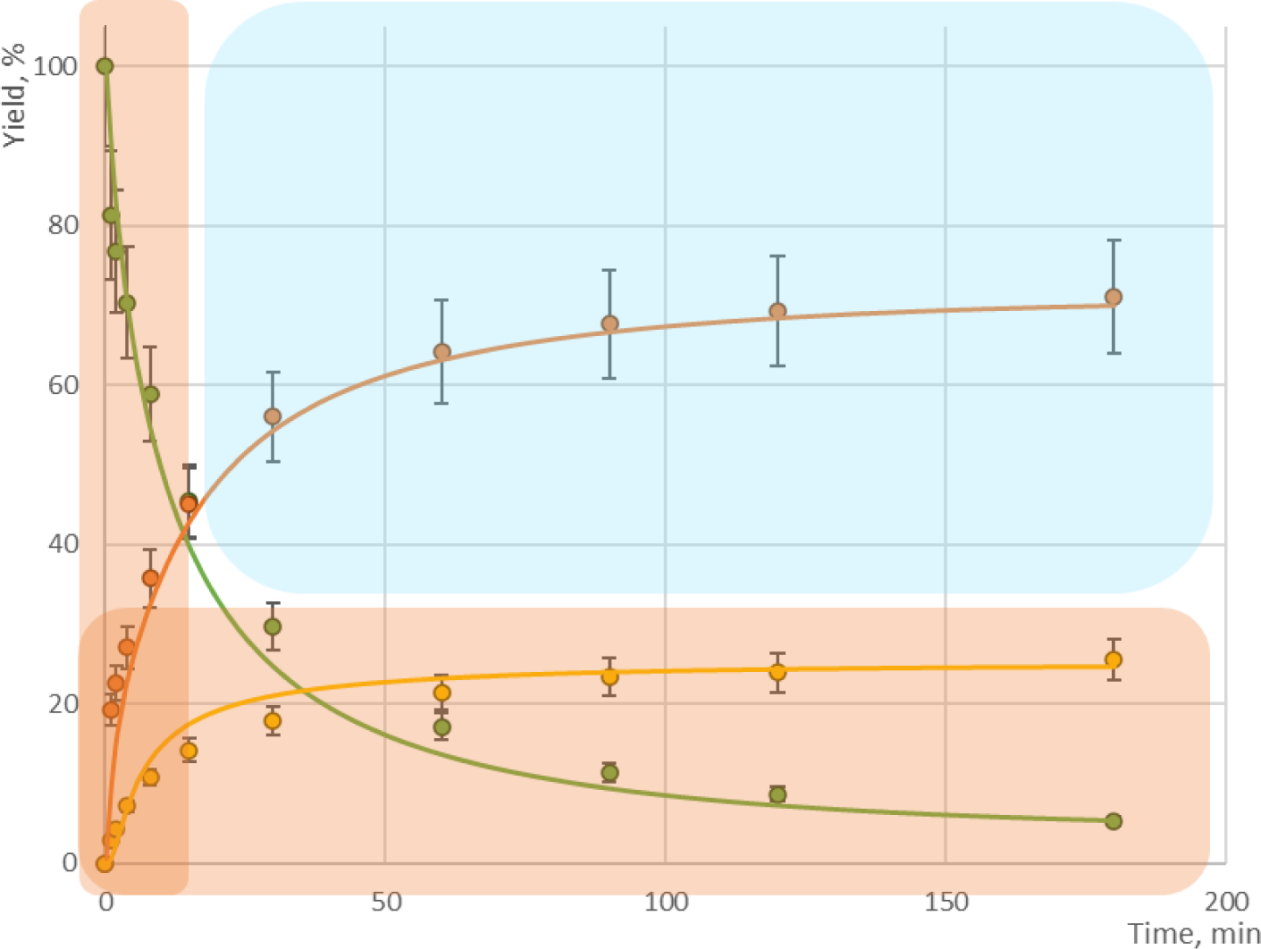
Diagram illustrating error magnitude in reaction time course plot. Areas shown in orange are sensitive to experimental errors, while the blue area is less affected.

The adequacy of a kinetic model can be considered as reproducing experimental results when all of the data points in focus are located satisfactorily close to the simulation curve in an overlaid plot. When evaluating the “distance” between experimental data and simulated curves, the former has scatter which typically follows a normal distribution from the true value. In the case of bias error, the cause is usually experimentally identifiable (temperature, stoichiometry, instrument calibration, etc.) and thus it is relatively suitable for statistical treatment. Meanwhile, the latter includes errors caused by model uncertainty, which are sometimes not even correlated to true values, unlike experimental data. Therefore, for model evaluation, simulated curves are better positioned at the center of the data to be tested since the major issue in modeling is to minimize model uncertainty, provided that experimental data are more related to true values. Moreover, since simulated data are continuous, it is beneficial to define a continuous error range to evaluate the distance to each experimental data point. Additionally, as discussed above, a larger error range needs to be considered in lower yield and early stages of reaction (Figure 3).

From this perspective, defining a nonlinear error range weighted by reaction progress and yield range in the model evaluation process is of considerable advantage. To implement a simple and practical error manipulation, some function that can describe exponential decays is desirable. Stirling’s exponential growth model is one good tool that exhibits this nature, eq 1.^21^ This function begins from *a* when *x* = 0, decays exponentially, and converges at a plateau of *a* – (*b*/*k*) when *b*, *k* < 0.
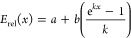
1

**Figure 4:**
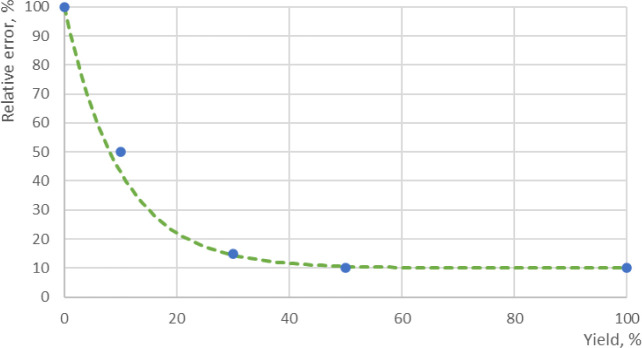
Fitted curve of eq 1 (green) on data point examples shown in Table 1 (blue circles). Parameters are *a* = 1, *b* = −9, and *k* = −10.

**Figure 5:**
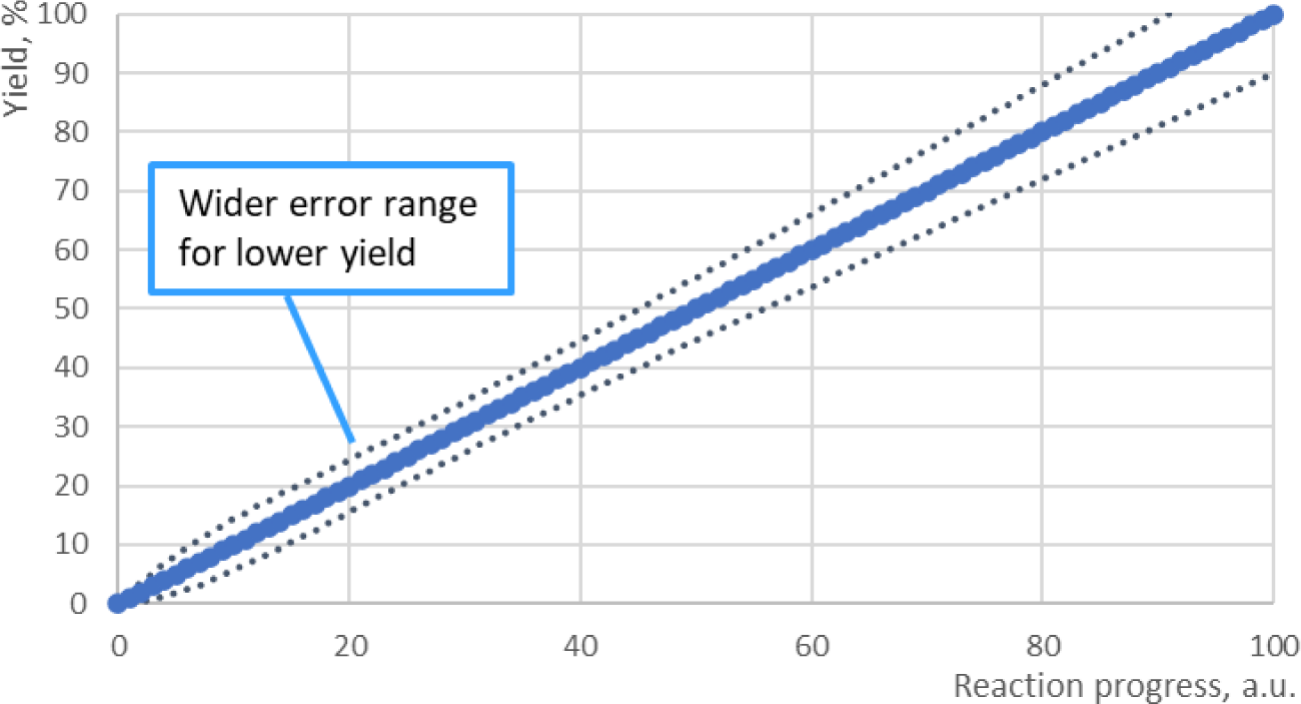
Weighted continuous relative error range, calculated based on parameters in Figure 4.

For example, in this discussion (although an approximate and empirical estimation), assume that relative experimental errors may be within 50% in lower yields such as 10% (i.e., expected error range of 5–15% at this point), while 10% in higher yield such as 50% (45–55% at this point), as shown in Table 1. In this case, when yield is set as *x* (1.0 for full conversion), curve regression using eq 1 showed a good fit with parameters *a* = 1, *b* = −9, and *k* = −10, respectively, as shown in Figure 4. Applying this function to each simulated yield gives a continuous, weighted relative error range as shown in Figure 5.

**Table 1:** Example of Weighted Relative Error for Each Stage of the Reaction

Simulated yield, %	Assumed relative error, %	Experimental yield range, %
0	(100)^a^	–
10	50	5–15
30	15	15–45
50	10	40–60
100	10	90–110

a Temporary value for fitting purpose as shown in Figure 4.

To numerically quantify model quality based on this idea, a weighted fitting index (WFI) can be introduced as shown in eq 2, where the absolute error is calculated as eq 3, and *n*, *E*_abs_, *Y*_s_, and *Y*_e_ stand for the number of data points, absolute error, simulated yield, and experimental yield, respectively. This index becomes <1 when the average distance between experimental and simulated yields is within the weighted error range defined by eq 1. This can thus be used as a good indicator for comparing models, as on minimizing this index, the simulation curves become closer to experimental data points (assuming exponential sampling intervals as discussed above). Additionally, changing the parameters of eq 1 can easily modify the threshold (weighting) as required. For example, when the modeling is focused on very low-level byproducts, i.e., < 1%, the error range shown above may not always be ideal in the target yield range. Also, if any validation of data precision is available, the error range can be narrowed to achieve more precise model evaluation.
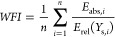
2
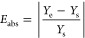
3

**Figure 6:**
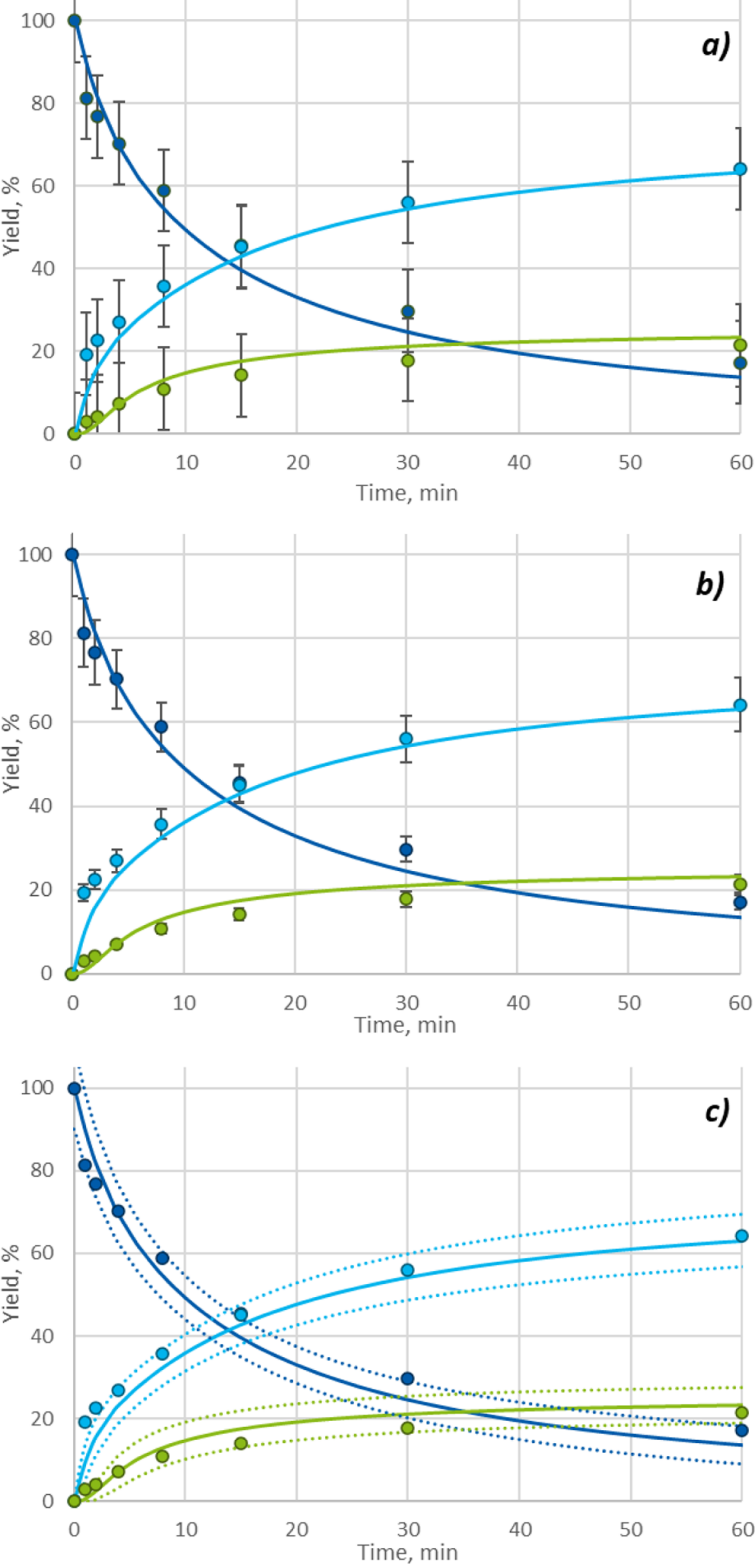
Illustrative example of the difference between error manipulation methods. (a) Absolute error bar (10%) centered on experimental values; (b) relative error bar (10%) centered on experimental values; (c) weighted error range (shown in Figures 4 and 5) centered on simulated curves.

Figure 6 summarizes an illustration of the differences between error manipulation methods. The absolute error bars (Figure 6a) overestimate in the lower yield range, such that 20% yield is “fitted” to a simulation result of 10%. On the other hand, standard relative error bars (Figure 6b) tend to be too strict to recognize that 18% yield is not fitted to 21% prediction. Thus, a simulation-centered error range (Figure 6c) is proposed to be advantageous in evaluating curve shape adequacy compared to simple error bars, as discussed above.

While this approach may also be utilized for model parameter optimization by minimizing the WFI, it is important to bear in mind that it merely indicates the distance between experimental data and the proposed hypothetical model in such cases. Thus, these types of calculations may not represent the most efficient means by themselves to explore the optimal model, i.e., the mechanism of the reaction. As mentioned in the previous sections, various experimental techniques, from extensive visual analysis to simple spiking experiments, should be employed in combination with chemical knowledge to effectively gather information to detect overlooked or misinterpreted elementary steps around the rds. Additionally, since the exponential sampling interval assumes a consistent reaction mechanism, careful consideration is required when a mechanism can possibly change during the reaction course, in which case alternative data collection strategies should be evaluated.

### Practical Application Example of Model Evaluation by the Proposed Model Validation Method

**Scheme 1:**
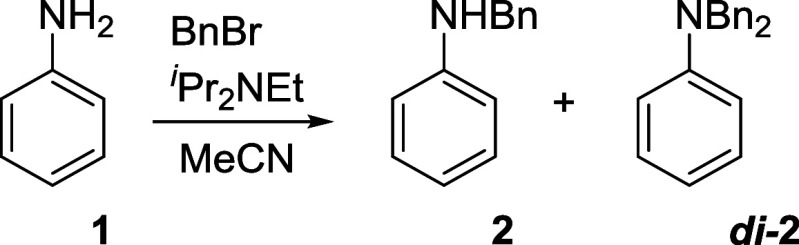
Benzylation of Unprotected Aniline

To confirm the capability of the weighted error range as a tool in kinetic model evaluation to achieve extrapolatable simulations in nonlinear, complex reactions, the benzylation of aniline was chosen as a simple test reaction (Scheme 1). This reaction was found to be suitable for testing the modeling process since it involves mono- and dibenzylation steps in a competing and consecutive manner, with no significant byproducts detected, and is easily analyzed by HPLC.

For collecting input data for the kinetic modeling, four experiments, which had different conditions in terms of stoichiometry balance and temperature, were conducted, and the reaction time courses were tracked by HPLC using biphenyl as an internal standard to calculate the conversion yields. In the initial modeling process of this reaction, solely S_N_2 or S_N_1 mechanisms were tested. Neither produced satisfactory curves in the self-reproducibility check nor exhibited extrapolability, suggesting the models were not sufficiently reasonable to describe the reaction mechanism. This was concluded even though, in some cases, the modeling tool showed “good” statistical indicators such as weighted sum-of-squares or confidence interval in the fitting program. Tables 2 and 3 show the fitting indices WFI calculated for the self-reproducibility check of both models using eq 2 (see the Supporting Information for more details and overlay plots for the self-reproducibility check).

**Table 2:** WFI Values for the S_N_2 Model

	WFI values for series of each species			
Exp.^a^	**1**	**2**	*di***–2**	Ave.
1	0.61	0.25	0.49	0.45
2	0.48	0.32	0.41	0.40
3	0.34	0.30	0.37	0.34
4	0.27	0.32	0.38	0.32

a Reaction conditions: DIPEA 1.5 eq, MeCN as solvent, 1) 40 °C, BnBr 1.2 eq; 2) 30 °C, BnBr 1.3 eq; 3) 50 °C, BnBr 1.1 eq; 4) 20 °C, BnBr 1.4 eq.

**Table 3:** WFI Values for S_N_1 Model

	WFI values for series of each species			
Exp.^a^	**1**	**2**	**di-2**	Ave.
1	0.35	0.19	0.33	0.29
2	0.23	0.22	0.22	0.22
3	0.23	0.23	0.25	0.24
4	0.18	0.28	0.23	0.23

a Reaction conditions: same as Table 2.

The WFI values suggest that the S_N_1 mechanism is a better fit than the S_N_2 mechanism, although the visual discrepancy in overlay plots still seems unsatisfactory, especially for extrapolability checks as shown in Figure 9. Additionally, the model of the S_N_2 mechanism with fractional orders exhibited a large discrepancy in extrapolation as well, even though self-reproducibility appeared improved compared to models with integer orders (see the Supporting Information).

**Figure 7:**
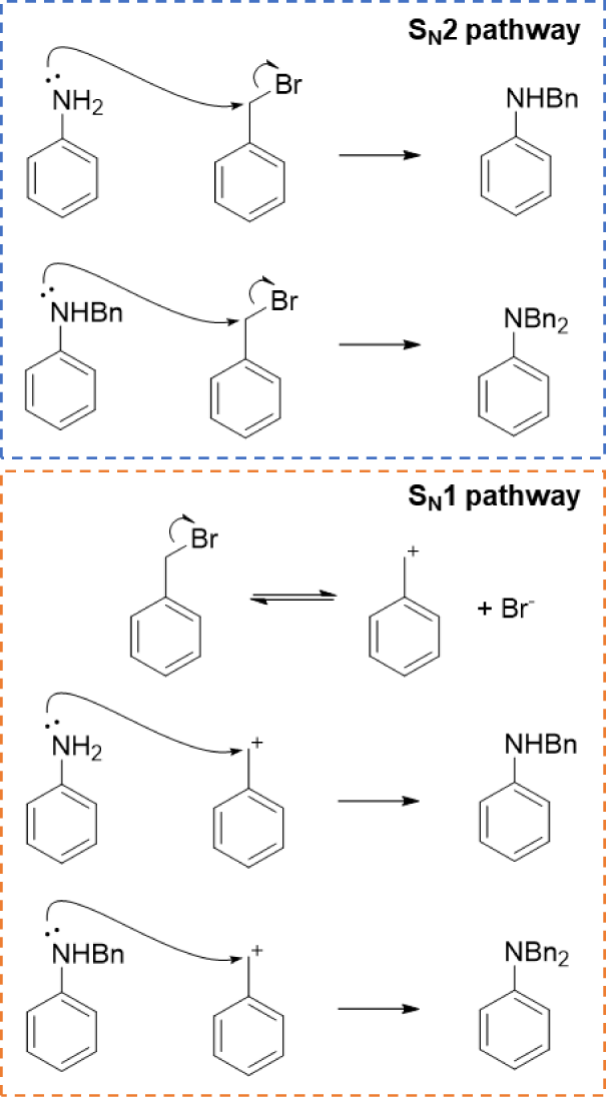
Elementary steps involved in the kinetic model of borderline mechanism for the mono- and dibenzylation of aniline.

**Figure 8:**
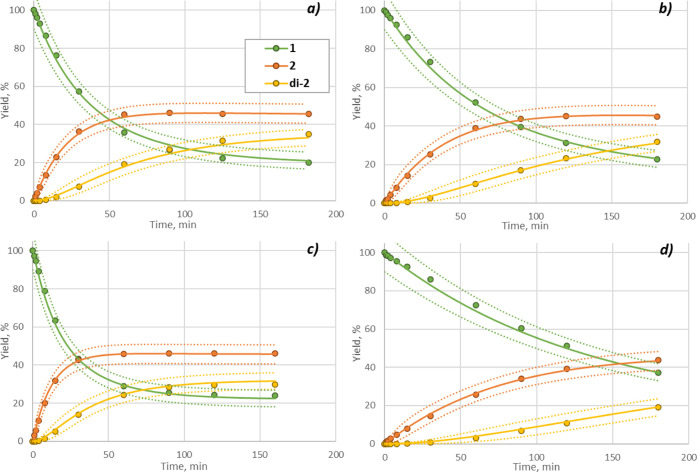
Overlay self-reproducibility plot of the borderline mechanism model. Filled circles: experimental data; lines: simulation; dotted lines: weighted error range. Reaction conditions: DIPEA 1.5 eq, MeCN as solvent, (a) 40 °C, BnBr 1.2 eq; (b) 30 °C, BnBr 1.3 eq; (c) 50 °C, BnBr 1.1 eq; (d) 20 °C, BnBr 1.4 eq. Yields are HPLC conversion yield.

Interestingly, as shown in Figure 8, when considering both S_N_1 and S_N_2 as competing reaction mechanisms (Figure 7) the model showed a better self-reproducibility of experimental values than either single mechanism model, i.e., only S_N_1 or S_N_2. In some cases, this type of S_N_ reaction is known to have a “borderline mechanism,” which proceeds through both S_N_1 and S_N_2 in a competing manner.^22−30^ It is notable that the kinetic modeling process itself can differentiate this unique type of reaction mechanism.

**Figure 9:**
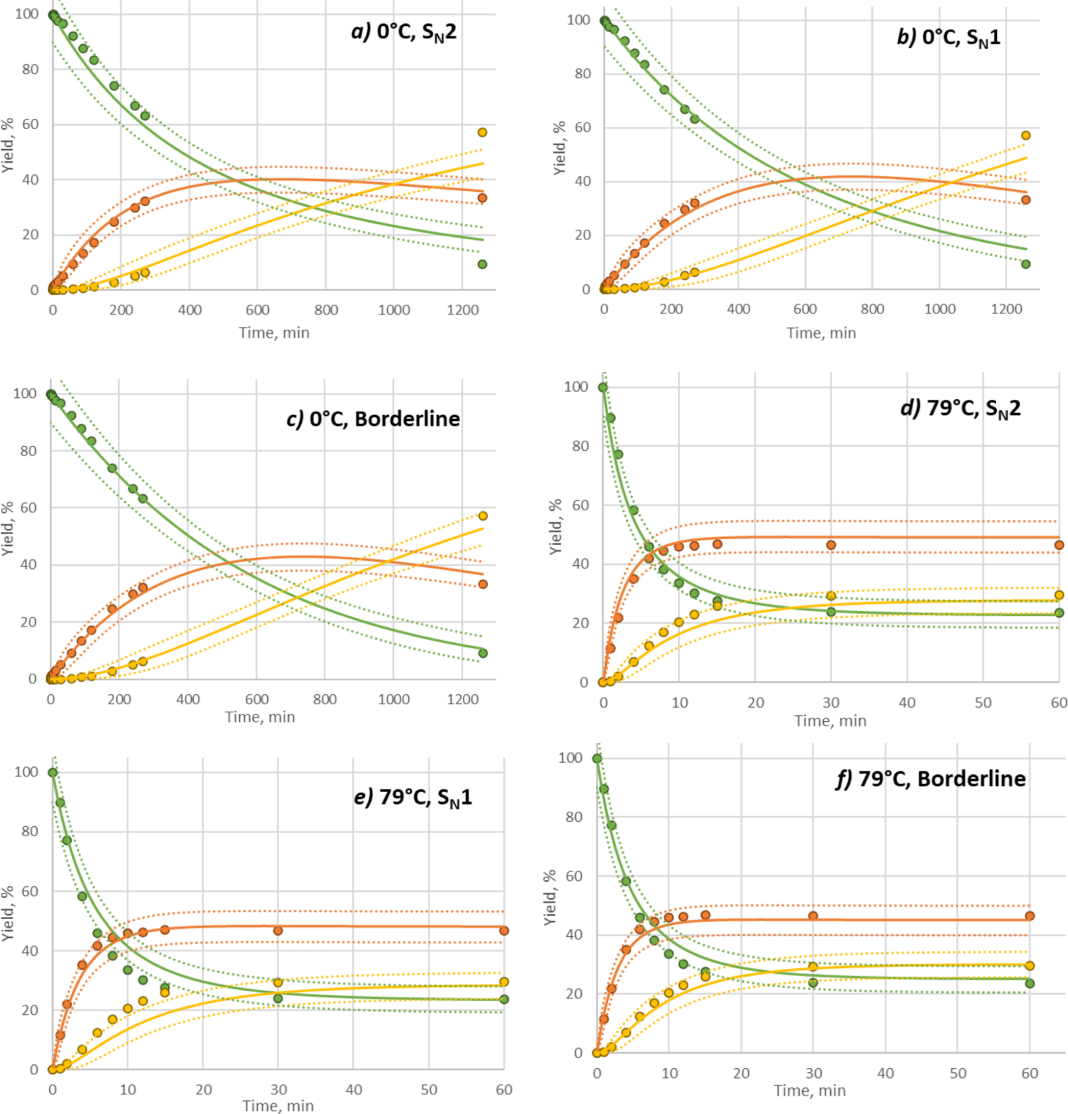
Extrapolability plot of simulations using each model. Experimental data outside of input range were separately collected and then compared with prediction results obtained by simulation using each kinetic model. Conditions: (a–c) 0 °C, BnBr 2.1 equiv; (d–f) 79 °C, BnBr 1.05 eq. Yields are HPLC conversion yield.

By employing the borderline mechanism, the fitting indices were further lowered, as shown in Table 4, suggesting the model more closely describes the actual reaction mechanism. Additionally, the resulting model showed good agreement with experimental results in self-reproducibility and extrapolability checks. Figure 9 summarizes the results of extrapolability checks at temperatures higher and lower than the input data range used for model building.

**Table 4:** WFI Values for the Borderline Model

	WFI values for series of each species			
Exp.^a^	**1**	**2**	**di-2**	Ave.
1	0.27	0.14	0.35	0.25
2	0.13	0.16	0.22	0.17
3	0.21	0.18	0.30	0.23
4	0.17	0.27	0.27	0.24

a Reaction conditions: same as Table 2.

**Figure 10:**
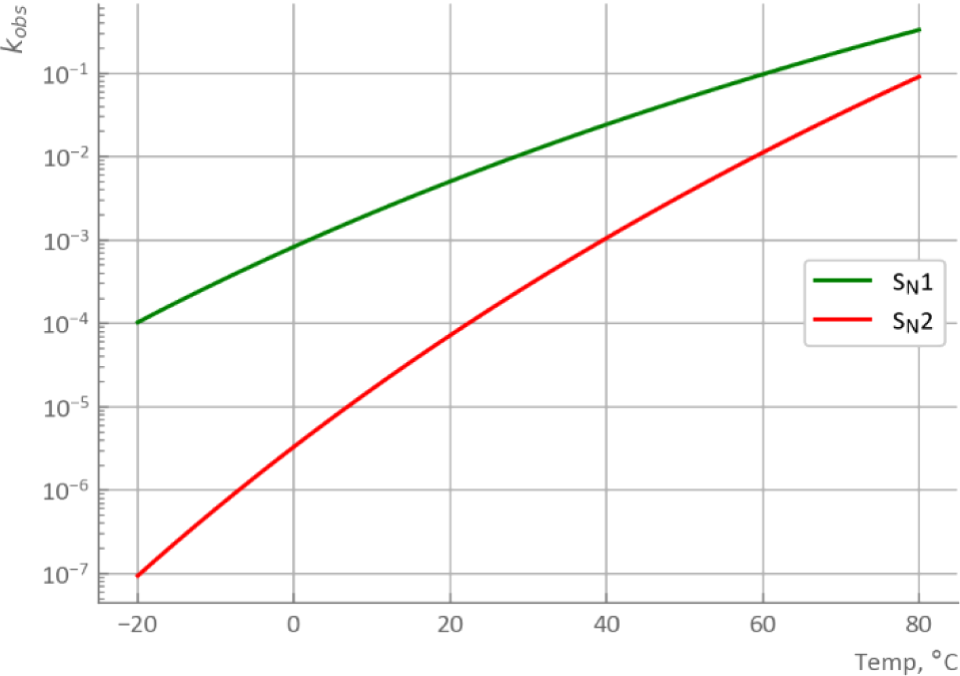
Temperature-dependent change of formal rate constants of S_N_1 [/s] and S_N_2 [L/mol.s].

**Figure 11:**
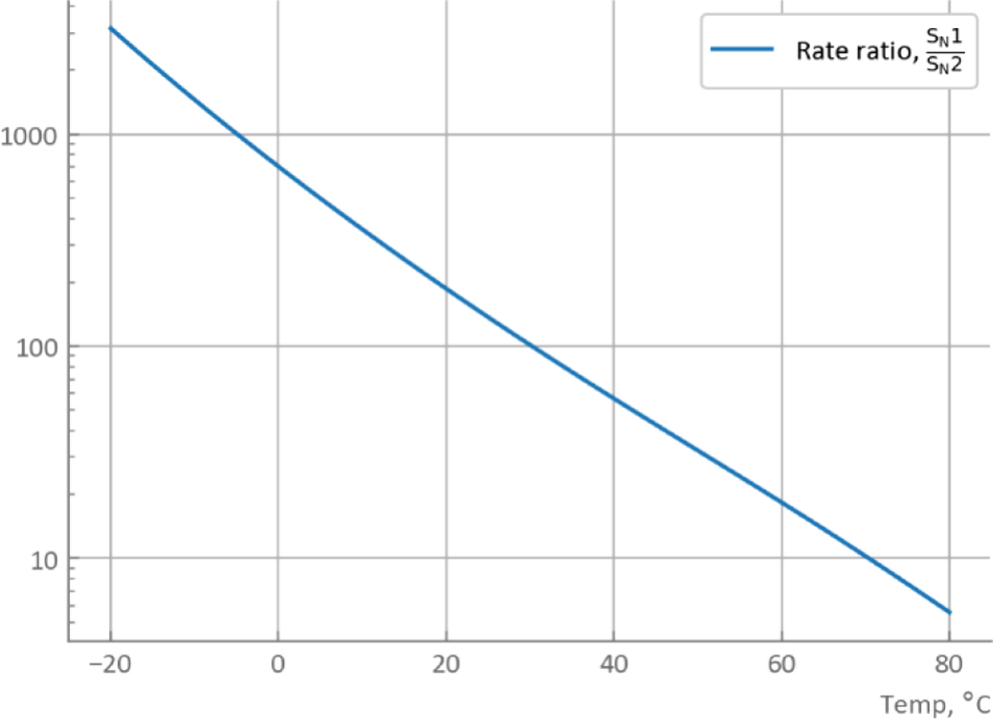
Calculated ratio of the reaction rate by simulation. Conditions: [**1**]_0_ 0.344 mmol/L, BnBr 1.1 equiv, as of 10 min after reaction start.

Furthermore, the results in Figure 9 suggested a temperature-dependent commitment ratio of mechanisms, since the S_N_1 model exhibited good reproducibility at lower temperatures, whereas the S_N_2 model performed better at higher temperatures. To confirm this, the temperature-dependent change of rate constants was calculated, as shown in Figure 10. The rate constant of the S_N_2 pathway changes dramatically with temperature, while that of the S_N_1 pathway is less sensitive. To be more precise, the ratio of reaction rates for fixed stoichiometry was calculated as an example (Figure 11) considering that these rate constants have different units for different orders. The temperature-dependent change in the ratio shows good agreement with the observation in Figure 9.

As discussed above, by introducing a weighted error range and fitting index in the modeling process, the model quality was effectively evaluated based on mechanistic understanding, and considerable improvement in the performance of extrapolative prediction by numerical simulation was obtained as expected. However, the selectivity of mono- versus dibenzylation was found to be limited to a low level even after thorough optimization using simulation. This means that it is difficult to obtain high monobenzylation selectivity based on this mechanism, i.e., similar reaction conditions using a mild base. Further investigation toward selective monobenzylation of unprotected aniline based on the mechanistic aspect will be reported in the near future.

## Conclusion

The concept and a practical example of the evaluation of a kinetic model in an experiment-based and mechanism-oriented manner have been described. In this methodology, a weighted continuous error range centered on simulated data is defined instead of using statistical indices such as error bars centered on experimental data. This approach provides a new aspect of model validation in kinetic modeling based on mechanistic understanding. Further applications, including reaction modeling directed by WFI, parameter estimation, and modification of this error range manipulation model, are currently under investigation.

## Supporting Information Available

Detailed experimental procedures, raw data, kinetic model parameters, calculation of WFI values, overlay self-reproducibility plots for S_N_2, S_N_1 models, and simulation data Model files (electronic, Reaction Lab/Dynochem binary files) will be provided as per reasonable request (PDF)
Practical methodology in kinetic modeling for complex reactions: weighted error manipulation to allow effective model evaluation in a borderline SN reaction (XLSX)

## Abbreviations

DIPEA: diisopropylethylamine
BnBr: benzyl bromide
MeCN: acetonitrile
rds: rate-determining step
