# Supplementary material for: Practical Methodology in Kinetic Modeling for Complex Reactions: Weighted Error Manipulation to Allow Effective Model Evaluation in a Borderline SN Reaction
Source: ACS Omega. 2025 Feb 24;10(9):9266–74. doi: 10.1021/acsomega.4c09609 (PMC11904694; doi:10.1021/acsomega.4c09609)
Supplement: Supplementary file 1 — ao4c09609_si_001.pdf [file ao4c09609_si_001.pdf]

Supporting Information

Practical methodology in kinetic modeling for complex reactions: weighted error manipulation to allow effective model evaluation in a borderline S_N reaction

Yuya Orito*,[a]

[a]*Process Technology Research Laboratories, Daiichi Sankyo Co., Ltd. 1-12-1 Shinomiya, Hiratsuka, Kanagawa 254-0014 Japan.*

Email: yuya.orito@daiichisankyo.com

Table of Contents

General. S2
- Materials and instruments. S2
- Chart drawings. S2
- Experimental details. S2
- HPLC condition. S2

Reaction modeling of aniline benzylation with $^{i}Pr_2NEt$. S3
- Typical experimental procedure. S3
- Modeling and simulation details. S5
  - Step-by-step procedure to utilize WFIs. S5
  - S_N2 single mechanism. S6
  - Example of fractional order model (based on S_N2 mechanism). S11
  - S_N1 single mechanism. S17
  - Borderline S_N mechanism. S21

General.

Materials and instruments.

All chemical materials, solvents were obtained from commercial sources (Sigma-Aldrich, Alfa Aesar, TCI, Kanto Chemical, or Wako Pure Chemical) and used without further purification. Dehydrated solvents were used within 2 days after freshly opened for all reactions. HPLC analysis were performed on Shimadzu LC-20 Prominence system fitted with UHPLC option and PDA (photodiode array) detector. Reaction modelings were performed by Reaction Lab and Dynochem (Scale-up Systems/Mettler-Toledo).

Chart drawings.

Charts are prepared by Microsoft Excel or Python scripts with matplotlib, using simulated and experimental data with appropriate calculations for HPLC quantification. Error range (dashed lines) are defined by using exponential growth Stirling model (Eq. 1), relative to simulated values as follows:

Upper error range: $Y_\mathrm{s} + Y_\mathrm{s} \times E_\mathrm{rel}(x)$; lower error range: $Y_\mathrm{s} - Y_\mathrm{s} \times E_\mathrm{rel}(x)$

Experimental details.

All reactions were performed using 100 mL glass separable reactor for EasyMax (Mettler-Toledo) with glass impeller, under inert gas (Ar or N_2) atmosphere, in EasyMax model 402 with Al vessel adaptor. Temperature was measured using digital thermometer with thermocouple and not corrected. Interval sampling for reaction time course tracking were performed using Nichiryo Nichipet Ex-Plus II volumetric mechanical pipette (20-200 μL) fitted with disposable long PP tip (Watson) and stopwatch device. HPLC peak identifications were based on retention time and UV/VIS spectra obtained by PDA detector, in comparison to authentic samples purchased from commercial sources. HPLC quantification were done using biphenyl as internal standard, in consideration with relative response factors, and then concentrations were calculated based on measured volume of reaction mixture.

HPLC condition.

All reaction monitoring analysis were performed by the same HPLC condition.

Column: Waters Acquity BEH C18 2.1 mm i.d. x 75 mm, 1.7 μm fitted with Ghost trap DS-HP (Shimadzu)

Temperature: 40°C

Solvent A: 10 mM $AcONH_4$

Solvent B: Acetonitrile

Gradient program:

0-3 min: B 40 ->90%

3-4 min: B 40%

Injection: 2 μL

Flow rate: 0.5 mL/min

Detection: UV 225 nm

T_R: Aniline (**1**) 0.67 min, BnBr 1.78 min, Benzylaniline (**2**) 2.08 min, Dibenzylaniline (***di*-2**) 3.17 min, Biphenyl 2.41 min. No other byproduct peaks more than 0.5% peak area detected.

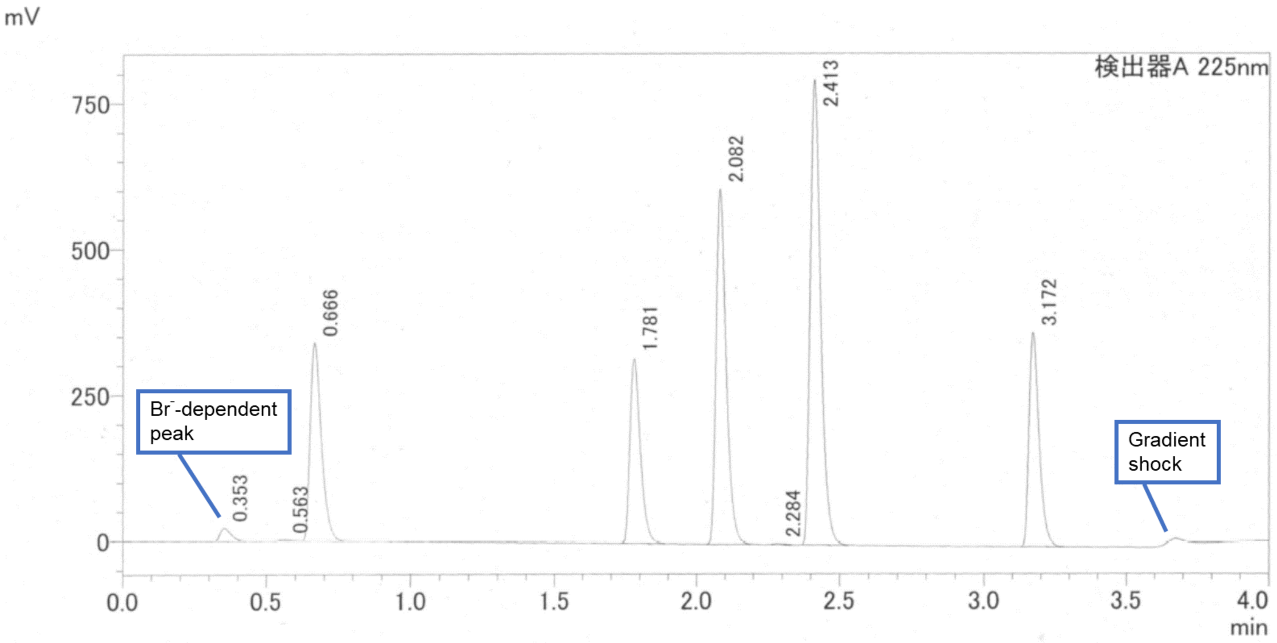

Figure S1. Typical HPLC chart (30°C, 180 min).

Reaction modeling of aniline benzylation with $^{i}Pr_2NEt$.

Typical experimental procedure.

To a 100 mL glass reactor was added MeCN (25 mL), aniline (1.00 g, 980 μL, 10.74 mmol), $^{i}Pr_2NEt$ (2.08 g, 16.11 mmol, 1.5 eq), and biphenyl (1.00 g, 6.48 mmol, 1.0 wt as internal standard), then the mixture was stirred at 40°C. To this mixture BnBr (2.57 g, 15.03 mmol, 1.5 eq) was added via syringe in one portion, then the reaction was monitored by HPLC to collect quantitative data. HPLC samples were prepared simply diluting the small aliquots (25 μL) of reaction mixture with 1 mL MeCN. Results are summarized in Table S1, S2.

Time, min	[**1**], mol/L	BnBr, mol/L	[**2**], mol/L	[***di*-2**], mol/L
**40°C, BnBr 1.2 eq, base 1.5 eq, Total volume 31.3 mL**				
0	0.34313	0.39940	0.00000	0.00000
1	0.33629	0.39193	0.00748	0.00000
2	0.32987	0.38625	0.01316	0.00000
4	0.31873	0.37484	0.02457	0.00000
8	0.29747	0.34973	0.04571	0.00198
15	0.26141	0.30639	0.07873	0.00714
30	0.19636	0.22294	0.12488	0.02579
60	0.12254	0.11231	0.15525	0.06593
90	0.09190	0.05663	0.15855	0.09211
125	0.07620	0.02593	0.15668	0.10839
182	0.06849	0.00330	0.15651	0.11980
**30°C, BnBr 1.3 eq, base 1.5 eq, Total volume 31.5 mL**				

0	0.34095	0.44324	0.00000	0.00000
1	0.33768	0.43807	0.00517	0.00000
2	0.33318	0.43572	0.00752	0.00000
4	0.32682	0.42865	0.01459	0.00000
8	0.31532	0.41433	0.02765	0.00063
15	0.29304	0.39021	0.04832	0.00235
30	0.25010	0.33843	0.08609	0.00936
60	0.17793	0.24350	0.13240	0.03367
90	0.13470	0.17745	0.14913	0.05833
120	0.10671	0.12996	0.15433	0.07947
180	0.07748	0.07304	0.15334	0.10843
**50ºC, BnBr 1.1 eq, base 1.5 eq, Total volume 31.2 mL**				
0	0.34423	0.37865	0.00000	0.00000
1	0.33450	0.36783	0.01082	0.00000
2	0.32551	0.35873	0.01992	0.00000
4	0.30711	0.33853	0.03761	0.00126
8	0.27160	0.29877	0.06932	0.00528
15	0.21851	0.23437	0.10942	0.01743
30	0.14913	0.13539	0.14707	0.04809
60	0.10003	0.05204	0.15825	0.08418
90	0.08759	0.02472	0.15943	0.09725
120	0.08382	0.01715	0.15914	0.10118
160	0.08238	0.01409	0.15924	0.10266
**20ºC, BnBr 1.4 eq, base 1.5 eq, Total volume 31.3 mL**				
0	0.33987	0.47582	0.00000	0.00000
1	0.33546	0.47201	0.00382	0.00000
2	0.33448	0.47019	0.00564	0.00000
4	0.33085	0.46674	0.00909	0.00000
8	0.32452	0.45992	0.01590	0.00000
15	0.31500	0.44759	0.02704	0.00060
30	0.29220	0.42157	0.04920	0.00253
60	0.24597	0.36763	0.08762	0.01029
90	0.20508	0.31456	0.11575	0.02276
120	0.17380	0.26927	0.13317	0.03669
180	0.12605	0.19667	0.14849	0.06533

Table S1. Quantitative reaction time courses of 4 different reaction condition for modeling. (Figure 8)

Time, min	[**1**], mol/L	BnBr, mol/L	[**2**], mol/L	[***di*-2**], mol/L
**0°C, BnBr 2.1 eq, base 1.5 eq, Total volume 32.5 mL**				
0	0.33046	0.69397	0.00000	0.00000
1	0.32972	0.69107	0.00290	0.00000
2	0.33037	0.69039	0.00358	0.00000
4	0.32795	0.68924	0.00473	0.00000
8	0.32642	0.68722	0.00675	0.00000
15	0.32247	0.68392	0.01005	0.00000
30	0.31971	0.67751	0.01646	0.00000
60	0.30486	0.66160	0.03053	0.00092
90	0.28982	0.64547	0.04419	0.00215
120	0.27601	0.62933	0.05677	0.00393
180	0.24482	0.59412	0.08116	0.00935
240	0.22111	0.56244	0.09847	0.01653
270	0.20879	0.54621	0.10624	0.02076
1260	0.03048	0.20448	0.11048	0.18951
**80°C, BnBr 1.05 eq, base 1.5 eq, Total volume 31.2 mL**				
0	0.344231	0.361442	0.000000	0.000000
1	0.308813	0.318284	0.040120	0.001519
2	0.265888	0.272270	0.075738	0.006717
4	0.200981	0.192969	0.121084	0.023694
6	0.158485	0.131967	0.143938	0.042769
8	0.131872	0.091646	0.153186	0.058305
10	0.115269	0.061914	0.158093	0.070718
12	0.104163	0.042987	0.159440	0.079508
15	0.094863	0.022188	0.161461	0.088896
30	0.082373	-0.001550	0.160592	0.101200
60	0.081559	-0.003349	0.160554	0.102119

Table S2. Quantitative reaction time courses for extrapolability check. (Figure 9)

Modeling and simulation details.

See attached Excel files for full simulation results.

Step-by-step procedure to utilize WFIs.

Figure S2 illustrates an example of implementation of the process utilizing WFIs. (*a*) Run the simulation with the model to be investigated. The output should be a series of time and calculated yield (Y_s) pairs. (*b*) For each point of Y_s, the weighted relative error (E_{rel}) can be calculated according to Eq. 1. The corresponding weighted error range is derived from the product of Y_s and E_{rel} (See 'Chart drawings' section above). For instance, when $Y_s = 0.700$ (70.0%),

E_{rel} = 0.101 (10.1%) results in a range from 0.771 (77.1%) to 0.629 (62.9%). Similarly, when Y_s = 0.100 (10.0%), E_{rel} = 0.431 (43.1%) results in a range from 0.143 (14.3%) to 0.057 (5.7%). The edges of the range (dashed lines) correspond to the experimental yield where WFI is 1.0. These error range calculations may be performed together with the simulation. (*c*) Compare the experimental yield (Y_e) with Y_s at the same time point. As shown in Eq. 3, the absolute error (E_{abs}) represents the normalized distance between Y_e and Y_s. Divide E_{abs} by the corresponding E_{rel} to obtain WFI for the data point (WFI(n) in the subsequent tables, corresponds to the ratio of yellow and green arrows). The WFI of the simulation curve is the average of WFI(n), as described in Eq. 2.

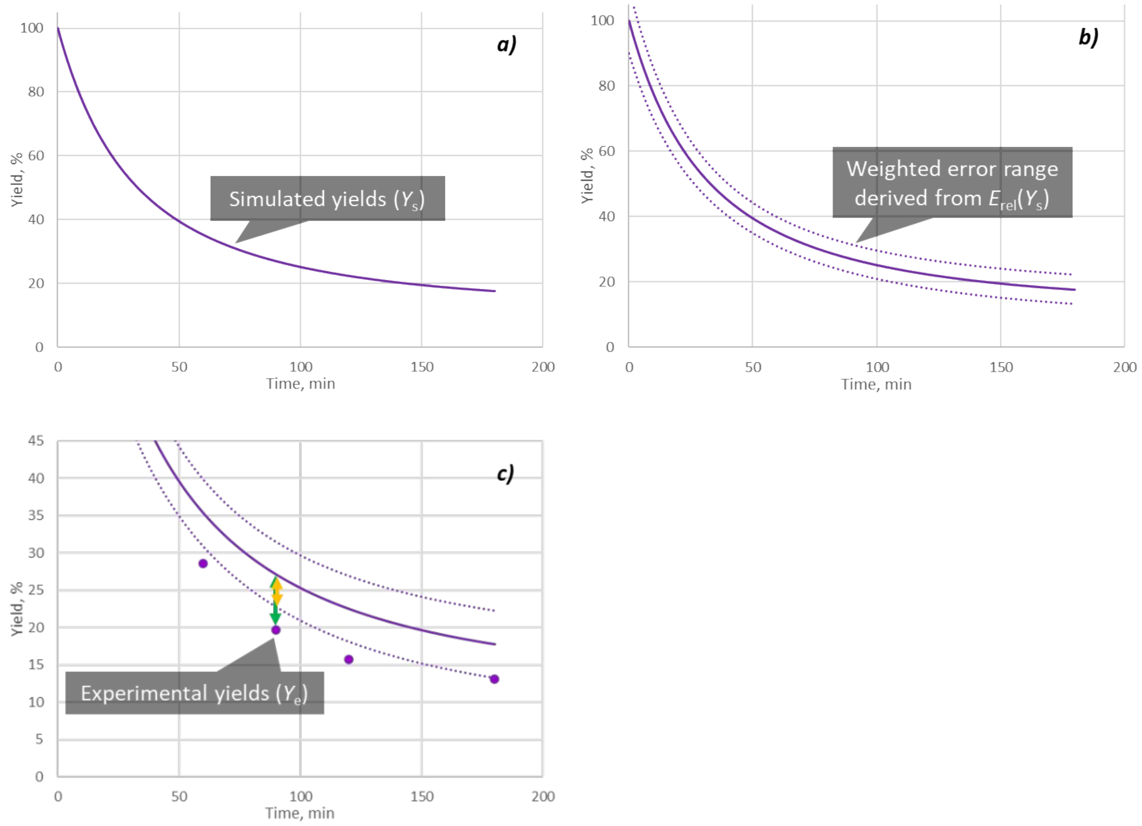

Figure S2. Illustrative examples of WFI calculations. *a*) Simulation results obtained from the model under investigation. *b*) Weighted error range calculation for each simulation data points. *c*) Compare the simulated and experimental yields to calculate WFI(n).

S_N2 single mechanism.

No.	Elementary reaction
1	**1** + BnBr → **2** + HBr
2	HBr + ^{i}Pr$_2$NEt ⇄ ^{i}Pr$_2$NEt·HBr
3	**2** + BnBr → ***di*-2** + HBr

No.	k (fwd), L/mol.min	Ea (fwd), kJ/mol	Keq		Ea (back), kJ/mol
1	0.0611	52.6			
2	1000	60	10	L/mol	60
3	0.0389	47.7			

Table S3. Model parameters of S_N2 model. Tref = 40℃ (for calculation of k)

Experiment	Series	Time, min	Y_e, %	Y_s, %	E_{abs}, %	E_{rel}, %	WFI(n)	WFI	Exp. Ave
40℃, BnBr 1.2 eq	**1**	0	100.0	100.0	0.0	10.0	0.00	0.61	0.45
		1	98.0	97.6	0.4	10.0	0.04		
		2	96.1	95.3	0.8	10.0	0.08		
		4	92.9	91.1	2.0	10.0	0.20		
		8	86.7	83.5	3.8	10.0	0.38		
		15	76.2	72.9	4.5	10.1	0.45		
		30	57.2	57.4	0.4	10.3	0.03		
		60	35.7	41.2	13.3	11.5	1.16		
		90	26.8	33.2	19.3	13.3	1.46		
		125	22.2	28.2	21.1	15.4	1.37		
		182	20.0	24.1	17.0	18.1	0.94		
	**2**	0	0.0	0.0	100.0	100.0	1.00	0.25	
		1	2.2	2.4	8.4	81.0	0.10		
		2	3.8	4.6	17.0	66.8	0.25		
		4	7.1	8.7	17.9	47.8	0.37		
		8	13.2	15.5	14.7	29.0	0.51		
		15	22.8	24.4	6.5	17.8	0.36		
		30	36.2	35.3	2.6	12.6	0.20		
		60	45.0	43.1	4.4	11.2	0.39		
		90	46.0	45.0	2.1	11.0	0.19		
		125	45.4	45.3	0.2	11.0	0.02		
		182	45.4	44.9	1.1	11.0	0.10		
	***di*-2**	0	0.0	0.0	100.0	100.0	1.00	0.49	
		1	0.0	0.0	100.0	99.8	1.00		
		2	0.0	0.1	100.0	99.4	1.01		
		4	0.0	0.3	100.0	97.6	1.02		
		8	0.5	0.9	41.6	92.0	0.45		
		15	2.0	2.7	26.5	78.8	0.34		
		30	7.1	7.3	2.4	53.4	0.05		
		60	18.2	15.7	15.7	28.7	0.55		
		90	25.4	21.8	16.6	20.2	0.82		

		125	29.9	26.5	12.6	16.3	0.77		
		182	33.0	31.1	6.4	14.0	0.45		
30℃, BnBr 1.3 eq	**1**	0	100.0	100.0	0.0	10.0	0.00	0.48	0.40
		1	99.0	98.6	0.4	10.0	0.04		
		2	97.7	97.3	0.4	10.0	0.04		
		4	95.9	94.7	1.2	10.0	0.12		
		8	92.5	89.9	2.9	10.0	0.29		
		15	85.9	82.5	4.2	10.0	0.42		
		30	73.4	69.8	5.0	10.1	0.50		
		61	52.2	52.9	1.3	10.5	0.12		
		90	39.5	43.2	8.5	11.2	0.76		
		120	31.3	36.6	14.4	12.3	1.17		
		180	22.7	28.6	20.5	15.2	1.36		
	**2**	0	0.0	0.0	100.0	100.0	1.00	0.32	
		1	1.5	1.4	11.1	88.5	0.13		
		2	2.2	2.7	17.8	78.8	0.23		
		4	4.3	5.2	17.7	63.5	0.28		
		8	8.1	9.8	16.7	43.9	0.38		
		15	14.2	16.4	13.6	27.4	0.49		
		30	25.3	26.6	4.9	16.3	0.30		
		61	38.9	37.4	3.9	12.1	0.32		
		90	43.8	41.6	5.2	11.4	0.45		
		120	45.3	43.5	4.3	11.2	0.38		
		180	45.0	44.1	2.1	11.1	0.19		
	***di*-2**	0	0.0	0.0	100.0	100.0	1.00	0.41	
		1	0.0	0.0	100.0	99.9	1.00		
		2	0.0	0.0	100.0	99.8	1.00		
		4	0.0	0.1	100.0	99.1	1.01		
		8	0.2	0.4	51.9	96.8	0.54		
		15	0.7	1.1	42.2	90.4	0.47		
		30	2.6	3.6	27.1	72.9	0.37		
		61	9.4	9.7	3.4	44.0	0.08		
		90	16.3	15.2	7.3	29.8	0.24		
		120	22.2	20.0	10.9	22.2	0.49		
		180	30.2	27.3	10.8	15.9	0.68		
50℃, BnBr 1.1 eq	**1**	0	100.0	100.0	0.0	10.0	0.00	0.34	0.34
		1	97.2	95.9	1.4	10.0	0.14		
		2	94.6	92.0	2.8	10.0	0.28		

		4	89.2	85.2	4.7	10.0	0.47		
		8	78.9	74.2	6.3	10.1	0.63		
		15	63.5	60.7	4.6	10.2	0.45		
		30	43.3	44.6	2.9	11.0	0.27		
		60	29.1	31.7	8.4	13.8	0.61		
		90	25.4	26.8	5.1	16.2	0.32		
		120	24.3	24.6	0.9	17.7	0.05		
		160	23.9	23.2	3.2	18.9	0.17		
	**2**	0	0.0	0.0	100.0	100.0	1.00	0.30	
		1	3.1	4.1	23.7	69.8	0.34		
		2	5.8	7.8	26.0	51.3	0.51		
		4	10.9	14.1	22.9	32.0	0.72		
		8	20.0	23.5	14.8	18.6	0.80		
		15	31.6	33.5	5.7	13.2	0.43		
		30	42.5	42.5	0.0	11.3	0.00		
		60	45.7	46.2	1.0	10.9	0.09		
		90	46.1	46.4	0.8	10.9	0.07		
		120	46.0	46.2	0.5	10.9	0.05		
		160	46.0	46.0	0.0	10.9	0.00		
	***di*-2**	0	0.0	0.0	100.0	100.0	1.00	0.37	
		1	0.0	0.1	100.0	99.5	1.00		
		2	0.0	0.2	100.0	98.2	1.02		
		4	0.3	0.7	52.7	93.8	0.56		
		8	1.4	2.3	38.4	81.6	0.47		
		15	4.7	5.8	19.3	60.6	0.32		
		30	12.8	12.9	0.5	34.8	0.01		
		60	22.5	22.1	1.7	19.9	0.08		
		90	26.0	26.8	3.1	16.2	0.19		
		120	27.0	29.2	7.6	14.8	0.51		
		160	27.4	30.8	11.1	14.1	0.78		
20℃, BnBr 1.4 eq	**1**	0	100.0	100.0	0.9	10.0	0.09	0.27	0.32
		1	98.7	99.3	1.5	10.0	0.15		
		2	98.4	98.6	1.1	10.0	0.11		
		4	97.3	97.1	0.7	10.0	0.07		
		8	95.5	94.4	0.2	10.0	0.02		
		15	92.7	90.0	2.0	10.0	0.20		
		30	86.0	81.6	4.4	10.0	0.44		
		60	72.4	68.5	4.7	10.1	0.47		

	90	60.3	58.8	1.7	10.3	0.16	
	120	51.1	51.4	1.5	10.5	0.15	
	180	37.1	41.2	10.8	11.5	0.94	
**2**	0	0.0	0.0	100.0	100.0	1.00	0.32
	1	1.1	0.7	52.4	93.7	0.56	
	2	1.6	1.4	13.6	87.9	0.15	
	4	2.7	2.8	6.8	77.8	0.09	
	8	4.7	5.5	15.6	62.1	0.25	
	15	7.9	9.6	18.8	44.3	0.42	
	30	14.4	17.1	16.5	26.3	0.63	
	60	25.6	27.3	7.1	15.8	0.45	
	90	33.9	33.6	0.3	13.1	0.02	
	120	39.0	37.5	2.8	12.1	0.23	
	180	43.4	41.4	4.0	11.4	0.35	
***di*-2**	0	0.0	0.0	100.0	100.0	1.00	0.38
	1	0.0	0.0	100.0	100.0	1.00	
	2	0.0	0.0	100.0	99.9	1.00	
	4	0.0	0.0	100.0	99.7	1.00	
	8	0.0	0.1	100.0	99.0	1.01	
	15	0.2	0.4	57.2	96.6	0.59	
	30	0.7	1.3	48.3	88.8	0.54	
	60	2.8	4.2	33.0	69.2	0.48	
	90	6.3	7.6	18.0	52.2	0.34	
	120	10.1	11.0	9.2	39.9	0.23	
	180	18.0	17.4	2.3	25.8	0.09	

Table S4. WFI calculations of S_N2 model.

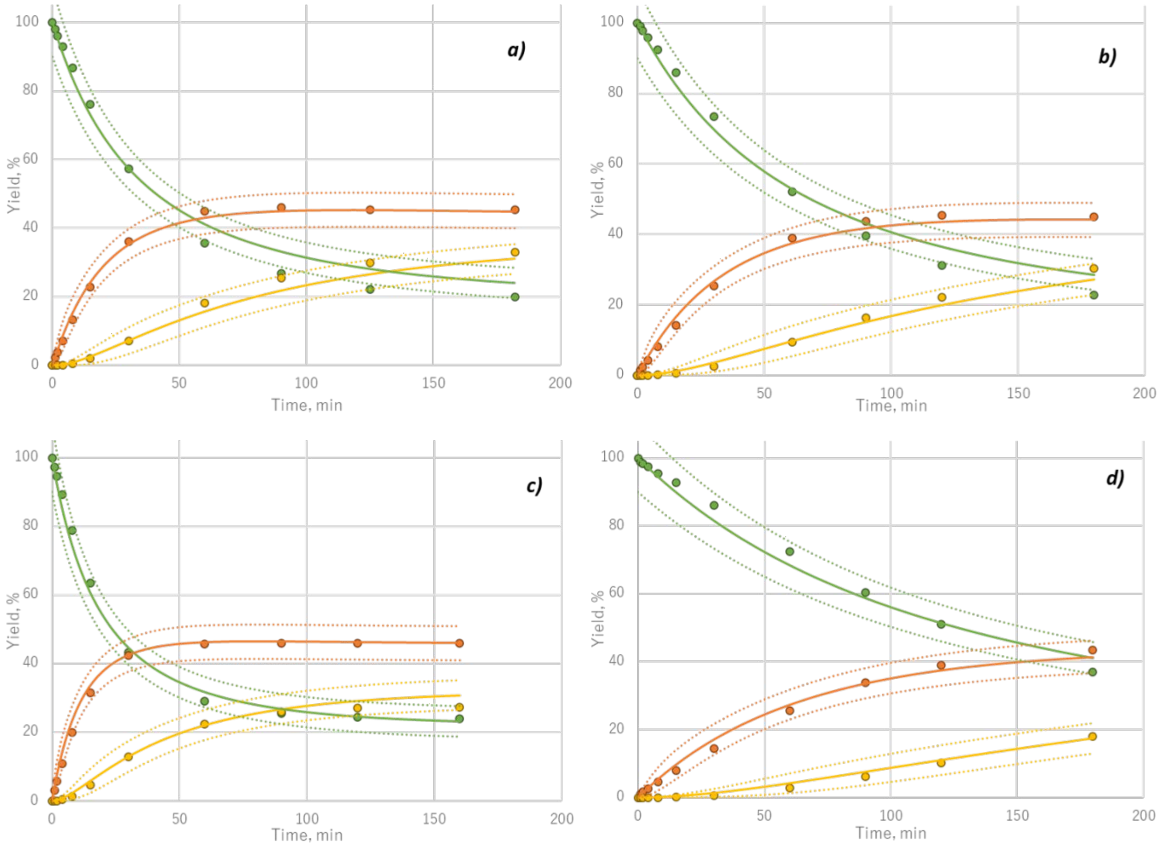

Figure S3. Overlay self-reproducibility plot of S_N2 mechanism model. Filled circles: experimental data; lines: simulation; dotted lines: weighted error range. Reaction conditions: DIPEA 1.5 eq, MeCN as solvent, *a*) 40°C, BnBr 1.2 eq; *b*) 30°C, BnBr 1.3 eq; *c*) 50°C, BnBr 1.1 eq; *d*) 20°C, BnBr 1.4 eq. Yields are HPLC conversion yield.

Example of fractional order model (based on S_N2 mechanism).

Fractional order fitting was performed using a function in Reaction Lab software. While multiple patterns of fractional orders were obtained depending on initial guess, one example model which gave minimum WFIs is described below.

No.	Elementary reaction
1	**1** + BnBr → **2** + HBr
2	HBr + ^{i}Pr$_2$NEt ⇄ ^{i}Pr$_2$NEt·HBr
3	**2** + BnBr → ***di*-2** + HBr

No.	*k* (fwd)	Ea (fwd), kJ/mol	Keq		Ea (back), kJ/mol	**1**, order	**2**, order	BnBr, order
1	1.887	52.6				0.16	-	1.29
2	1000	60	1000	L/mol	60			
3	1.429	47.7				-	1.62	1.18

Table S5. Model parameters of S_N2 model with fractional orders. Tref = 40℃ (for calculation of *k*)

Experiment	Series	Time, min	Y_e, %	Y_s, %	E_{abs}, %	E_{rel}, %	WFI(n)	WFI	Exp Ave
40℃, BnBr 1.2 eq	**1**	0	100.0	100.0	0.0	10.0	0.0	0.38	0.33
		1	98.0	98.1	0.1	10.0	0.0		
		2	96.1	96.3	0.1	10.0	0.0		
		4	92.9	92.7	0.2	10.0	0.0		
		8	86.7	86.1	0.7	10.0	0.1		
		15	76.2	76.0	0.2	10.0	0.0		
		30	57.2	59.5	3.8	10.2	0.4		
		60	35.7	40.5	11.8	11.6	1.0		
		90	26.8	31.3	14.3	14.0	1.0		
		125	22.2	25.8	13.9	16.8	0.8		
		182	20.0	21.8	8.6	20.1	0.4		
	**2**	0	0.0	0.0	100.0	100.0	1.0	0.19	
		1	2.2	1.9	15.1	84.6	0.2		
		2	3.8	3.7	2.7	72.1	0.0		
		4	7.1	7.2	1.1	53.8	0.0		
		8	13.2	13.5	1.7	33.4	0.0		
		15	22.8	22.3	2.3	19.7	0.1		
		30	36.2	34.2	5.9	13.0	0.5		
		60	45.0	43.4	3.7	11.2	0.3		
		90	46.0	46.1	0.3	10.9	0.0		
		125	45.4	47.0	3.4	10.8	0.3		
		182	45.4	47.3	4.1	10.8	0.4		
	***di*-2**	0	0.0	0.0	100.0	100.0	1.0	0.42	
		1	0.0	0.0	100.0	100.0	1.0		
		2	0.0	0.0	100.0	99.9	1.0		
		4	0.0	0.1	100.0	99.3	1.0		
		8	0.5	0.4	32.4	96.4	0.3		
		15	2.0	1.7	16.2	86.0	0.2		
		30	7.1	6.3	12.2	57.7	0.2		
		60	18.2	16.1	13.0	28.0	0.5		
		90	25.4	22.7	12.1	19.3	0.6		
		125	29.9	27.2	9.9	15.9	0.6		
		182	33.0	30.9	7.0	14.1	0.5		
30℃, BnBr 1.3 eq	**1**	0	100.0	100.0	0.0	10.0	0.0	0.18	0.17
		1	99.0	98.9	0.2	10.0	0.0		
		2	97.7	97.8	0.1	10.0	0.0		

		4	95.9	95.6	0.3	10.0	0.0		
		8	92.5	91.4	1.1	10.0	0.1		
		15	85.9	84.6	1.5	10.0	0.2		
		30	73.4	72.0	1.8	10.1	0.2		
		61	52.2	53.0	1.6	10.4	0.2		
		90	39.5	41.4	4.5	11.4	0.4		
		120	31.3	33.3	6.1	13.2	0.5		
		180	22.7	24.0	5.4	18.1	0.3		
	**2**	0	0.0	0.0	100.0	100.0	1.0	0.16	
		1	1.5	1.1	35.7	90.5	0.4		
		2	2.2	2.2	0.6	82.1	0.0		
		4	4.3	4.4	2.0	68.1	0.0		
		8	8.1	8.4	3.8	48.7	0.1		
		15	14.2	14.8	4.1	30.5	0.1		
		30	25.3	25.4	0.3	17.1	0.0		
		61	38.9	37.6	3.4	12.1	0.3		
		90	43.8	42.7	2.5	11.3	0.2		
		120	45.3	45.2	0.2	11.0	0.0		
		180	45.0	47.0	4.1	10.8	0.4		
	***di*-2**	0	0.0	0.0	100.0	100.0	1.0	0.17	
		1	0.0	0.0	100.0	100.0	1.0		
		2	0.0	0.0	100.0	100.0	1.0		
		4	0.0	0.0	100.0	99.8	1.0		
		8	0.2	0.1	43.6	98.9	0.4		
		15	0.7	0.6	17.7	95.1	0.2		
		30	2.6	2.6	0.9	79.5	0.0		
		61	9.4	9.4	0.2	45.3	0.0		
		90	16.3	15.9	2.4	28.4	0.1		
		120	22.2	21.5	3.3	20.5	0.2		
		180	30.2	29.0	4.2	14.9	0.3		
50℃, BnBr 1.1 eq	**1**	0	100.0	100.0	0.0	10.0	0.0	0.18	0.19
		1	97.2	96.8	0.4	10.0	0.0		
		2	94.6	93.6	1.0	10.0	0.1		
		4	89.2	87.8	1.6	10.0	0.2		
		8	78.9	77.6	1.6	10.0	0.2		
		15	63.5	63.7	0.3	10.2	0.0		
		30	43.3	45.3	4.4	11.0	0.4		
		60	29.1	30.5	4.8	14.3	0.3		

		90	25.4	25.5	0.4	17.0	0.0		
		120	24.3	23.5	3.6	18.6	0.2		
		160	23.9	22.3	7.1	19.6	0.4		
	**2**	0	0.0	0.0	100.0	100.0	1.0	0.19	
		1	3.1	3.2	3.5	75.1	0.0		
		2	5.8	6.3	8.7	57.9	0.2		
		4	10.9	11.9	8.6	37.4	0.2		
		8	20.0	21.0	4.7	21.0	0.2		
		15	31.6	31.7	0.2	13.8	0.0		
		30	42.5	42.0	1.3	11.4	0.1		
		60	45.7	46.8	2.3	10.8	0.2		
		90	46.1	47.6	3.2	10.8	0.3		
		120	46.0	47.7	3.6	10.8	0.3		
		160	46.0	47.6	3.4	10.8	0.3		
	***di*-2**	0	0.0	0.0	100.0	100.0	1.0	0.20	
		1	0.0	0.0	100.0	99.9	1.0		
		2	0.0	0.1	100.0	99.5	1.0		
		4	0.3	0.3	19.7	97.5	0.2		
		8	1.4	1.4	3.9	88.6	0.0		
		15	4.7	4.7	0.1	66.5	0.0		
		30	12.8	12.8	0.6	35.1	0.0		
		60	22.5	22.7	1.0	19.3	0.1		
		90	26.0	26.9	3.5	16.1	0.2		
		120	27.0	28.8	6.3	15.0	0.4		
		160	27.4	30.0	8.8	14.5	0.6		
20℃, BnBr 1.4 eq	**1**	0	100.0	100.0	0.9	10.0	0.1	0.13	0.18
		1	98.7	99.4	1.6	10.0	0.2		
		2	98.4	98.8	1.3	10.0	0.1		
		4	97.3	97.6	1.2	10.0	0.1		
		8	95.5	95.3	0.8	10.0	0.1		
		15	92.7	91.4	0.4	10.0	0.0		
		30	86.0	83.6	1.9	10.0	0.2		
		60	72.4	70.2	2.1	10.1	0.2		
		90	60.3	59.4	0.6	10.2	0.1		
		120	51.1	50.7	0.1	10.6	0.0		
		180	37.1	38.1	3.7	12.0	0.3		
	**2**	0	0.0	0.0	100.0	100.0	1.0	0.23	
		1	1.1	0.6	84.1	94.8	0.9		

	2	1.6	1.2	36.5	89.8	0.4	
	4	2.7	2.4	10.9	81.0	0.1	
	8	4.7	4.7	1.3	66.4	0.0	
	15	7.9	8.5	7.6	48.6	0.2	
	30	14.4	15.7	9.2	28.7	0.3	
	60	25.6	26.6	4.4	16.3	0.3	
	90	33.9	33.7	0.4	13.1	0.0	
	120	39.0	38.2	0.9	12.0	0.1	
	180	43.4	43.1	0.0	11.2	0.0	
***di*-2**	0	0.0	0.0	100.0	100.0	1.0	0.18
	1	0.0	0.0	100.0	100.0	1.0	
	2	0.0	0.0	100.0	100.0	1.0	
	4	0.0	0.0	100.0	100.0	1.0	
	8	0.0	0.0	100.0	99.8	1.0	
	15	0.2	0.1	23.5	98.8	0.2	
	30	0.7	0.7	2.1	93.9	0.0	
	60	2.8	3.2	12.9	75.2	0.2	
	90	6.3	6.9	10.4	55.0	0.2	
	120	10.1	11.1	9.4	39.8	0.2	
	180	18.0	18.8	5.2	23.7	0.2	

Table S6. WFI calculations of S_N2 model with fractional orders.

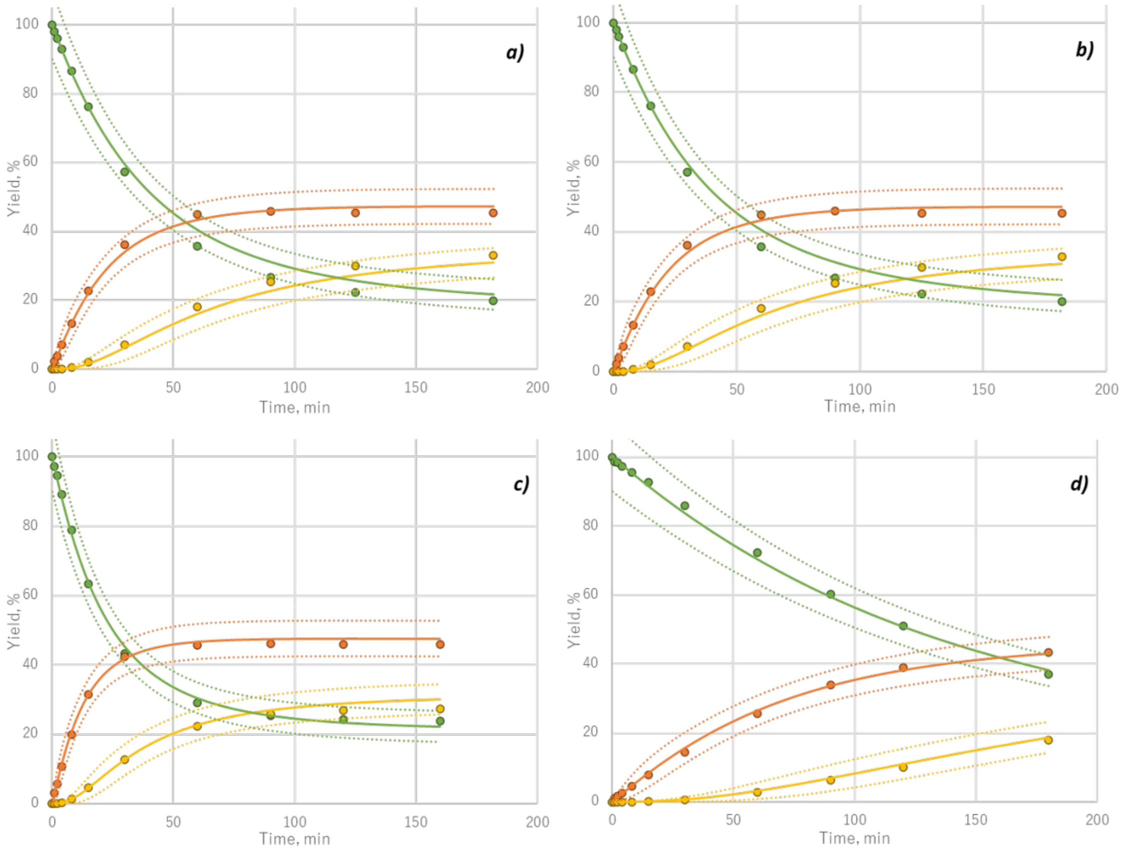

Figure S4. Overlay self-reproducibility plot of S_N2 mechanism model with fractional orders. Filled circles: experimental data; lines: simulation; dotted lines: weighted error range. Reaction conditions: DIPEA 1.5 eq, MeCN as solvent, *a*) 40°C, BnBr 1.2 eq; *b*) 30°C, BnBr 1.3 eq; *c*) 50°C, BnBr 1.1 eq; *d*) 20°C, BnBr 1.4 eq. Yields are HPLC conversion yield.

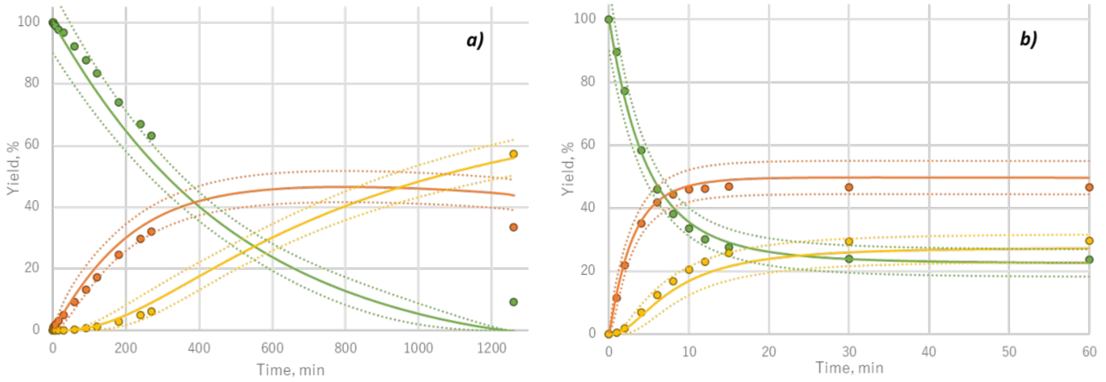

Figure S5. Extrapolability test plot of S_N2 mechanism model with fractional orders. Experimental data outside of input range were separately collected, and then compared with prediction result obtained by simulation. Conditions: *a*) 79°C, BnBr 1.05 eq; *b*) 0°C, BnBr 2.1 eq. Yields are HPLC conversion yield.

S_N1 single mechanism.

No.	Elementary reaction
1	HBr + $^{i}Pr_2NEt$ ⇄ $^{i}Pr_2NEt{\cdot}HBr$
2	BnBr ⇄ Int
3	Int + **1** → **2** + HBr
4	Int + **2** → ***di*-2** + HBr

*Int = 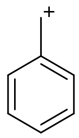

No.	*k* (fwd), L/mol.min	Ea (fwd), kJ/mol	Keq		Ea (back), kJ/mol
2	1000	60	1000	L/mol	60
4	0.0243	60	0.01	-	45
5	31.68	3.3			
6	19.94	0			

Table S7. Model parameters of S_N1 model. Tref = 40℃ (for calculation of *k*)

Experiment	Series	Time, min	Y_e, %	Y_s, %	E_{abs}, %	E_{rel}, %	WFI(*n*)	WFI	Exp Ave
40℃, BnBr 1.2 eq	**1**	0	100.0	100.0	0.0	10.0	0.00	0.35	0.29
		1	98.0	97.9	0.1	10.0	0.01		
		2	96.1	95.7	0.4	10.0	0.04		
		4	92.9	91.5	1.5	10.0	0.15		
		8	86.7	84.0	3.2	10.0	0.31		
		15	76.2	73.1	4.2	10.1	0.42		
		30	57.2	56.4	1.4	10.3	0.14		
		60	35.7	38.5	7.2	11.9	0.60		
		90	26.8	30.0	10.7	14.5	0.74		
		125	22.2	25.1	11.6	17.3	0.67		
		182	20.0	21.8	8.3	20.2	0.41		
	**2**	0	0.0	0.0	100.0	100.0	1.00	0.19	
		1	2.2	2.1	4.5	83.1	0.05		
		2	3.8	4.2	9.4	68.9	0.14		
		4	7.2	8.2	12.9	49.6	0.26		
		8	13.3	15.1	11.7	29.9	0.39		
		15	22.9	24.3	5.5	17.9	0.31		
		30	36.4	36.0	1.2	12.5	0.10		
		60	45.2	44.1	2.6	11.1	0.23		
		90	46.2	45.5	1.5	10.9	0.14		
		125	45.7	45.3	0.8	11.0	0.07		
		182	45.6	44.6	2.2	11.0	0.20		

	***di*-2**	0	0.0	0.0	100.0	100.0	1.00	0.33	
		1	0.0	0.0	100.0	99.9	1.00		
		2	0.0	0.1	100.0	99.5	1.01		
		4	0.0	0.2	100.0	97.9	1.02		
		8	0.6	0.9	33.3	92.5	0.36		
		15	2.1	2.6	20.3	79.3	0.26		
		30	7.5	7.6	1.2	52.1	0.02		
		60	19.2	17.4	10.2	25.7	0.40		
		90	26.8	24.5	9.7	17.8	0.54		
		125	31.6	29.6	6.9	14.7	0.47		
		182	34.9	33.6	3.9	13.1	0.30		
30℃, BnBr 1.3 eq	**1**	0	100.0	100.0	0.0	10.0	0.00	0.23	0.22
		1	99.0	98.8	0.2	10.0	0.02		
		2	97.7	97.6	0.2	10.0	0.02		
		4	95.9	95.1	0.8	10.0	0.08		
		8	92.5	90.4	2.3	10.0	0.23		
		15	85.9	83.1	3.5	10.0	0.35		
		30	73.4	70.0	4.8	10.1	0.47		
		60	52.2	52.0	0.5	10.5	0.04		
		90	39.5	40.6	2.6	11.6	0.23		
		120	31.3	33.1	5.4	13.3	0.41		
		180	22.7	24.6	7.5	17.7	0.42		
	**2**	0	0.0	0.0	100.0	100.0	1.00	0.22	
		1	1.5	1.2	29.0	90.0	0.32		
		2	2.2	2.4	9.2	80.6	0.11		
		4	4.3	4.8	11.5	65.5	0.17		
		8	8.1	9.3	12.4	45.7	0.27		
		15	14.2	15.9	11.0	28.3	0.39		
		30	25.2	26.6	4.9	16.3	0.30		
		60	38.8	38.2	1.8	12.0	0.15		
		90	43.7	42.9	1.9	11.2	0.17		
		120	45.3	44.5	1.7	11.0	0.15		
		180	45.0	44.3	1.5	11.1	0.13		
	***di*-2**	0	0.0	0.0	100.0	100.0	1.00	0.22	
		1	0.0	0.0	100.0	100.0	1.00		
		2	0.0	0.0	100.0	99.8	1.00		
		4	0.0	0.1	100.0	99.3	1.01		
		8	0.2	0.3	41.5	97.2	0.43		

		15	0.7	1.0	32.6	91.3	0.36		
		30	2.7	3.4	20.1	73.8	0.27		
		60	9.9	9.9	0.2	43.5	0.00		
		90	17.1	16.5	3.5	27.2	0.13		
		120	23.3	22.4	4.2	19.6	0.21		
		180	31.8	31.1	2.2	14.0	0.15		
50℃, BnBr 1.1 eq	**1**	0	100.0	100.0	0.0	10.0	0.00	0.23	0.24
		1	97.2	96.4	0.8	10.0	0.08		
		2	94.6	92.7	2.0	10.0	0.20		
		4	89.2	86.1	3.6	10.0	0.36		
		8	78.9	75.1	5.1	10.0	0.51		
		15	63.5	60.9	4.3	10.2	0.42		
		30	43.3	43.3	0.0	11.2	0.00		
		60	29.1	29.6	1.9	14.6	0.13		
		90	25.4	25.1	1.3	17.3	0.08		
		120	24.3	23.4	4.1	18.7	0.22		
		160	23.9	22.6	6.1	19.4	0.31		
	**2**	0	0.0	0.0	100.0	100.0	1.00	0.23	
		1	3.1	3.6	12.1	72.9	0.17		
		2	5.8	7.1	18.3	54.3	0.34		
		4	10.9	13.3	17.5	33.9	0.52		
		8	20.1	22.8	11.7	19.2	0.61		
		15	31.8	33.4	4.8	13.2	0.36		
		30	42.7	42.9	0.5	11.2	0.04		
		60	46.0	46.3	0.6	10.9	0.06		
		90	46.3	46.2	0.4	10.9	0.03		
		120	46.2	45.9	0.7	10.9	0.07		
		160	46.3	45.7	1.2	10.9	0.11		
	***di*-2**	0	0.0	0.0	100.0	100.0	1.00	0.25	
		1	0.0	0.0	100.0	99.6	1.00		
		2	0.0	0.2	100.0	98.5	1.01		
		4	0.4	0.6	41.5	94.6	0.44		
		8	1.5	2.1	28.3	82.7	0.34		
		15	5.1	5.8	12.0	60.6	0.20		
		30	14.0	13.7	1.7	32.8	0.05		
		60	24.5	24.1	1.5	18.1	0.08		
		90	28.3	28.7	1.7	15.1	0.11		
		120	29.4	30.7	4.3	14.2	0.30		

		160	29.8	31.7	6.0	13.8	0.43		
20°C, BnBr 1.4 eq	**1**	0	100.0	100.0	0.0	10.0	0.00	0.18	0.23
		1	98.7	99.4	0.7	10.0	0.07		
		2	98.4	98.8	0.4	10.0	0.04		
		4	97.3	97.5	0.1	10.0	0.01		
		8	95.5	95.0	0.5	10.0	0.05		
		15	92.7	90.9	2.0	10.0	0.20		
		30	86.0	82.8	3.8	10.0	0.38		
		60	72.4	69.6	4.0	10.1	0.40		
		90	60.3	59.2	1.9	10.2	0.18		
		120	51.1	51.0	0.2	10.5	0.02		
		180	37.1	39.2	5.4	11.8	0.46		
	**2**	0	0.0	0.0	100.0	100.0	1.00	0.28	
		1	1.1	0.6	90.2	94.8	0.95		
		2	1.7	1.2	34.5	89.6	0.39		
		4	2.7	2.5	7.3	80.1	0.09		
		8	4.7	4.9	4.7	65.1	0.07		
		15	8.0	8.8	9.9	47.2	0.21		
		30	14.5	16.1	9.9	28.1	0.35		
		60	25.8	26.7	3.5	16.2	0.22		
		90	34.1	33.7	1.0	13.1	0.08		
		120	39.2	38.2	2.5	12.0	0.21		
		180	43.7	42.7	2.4	11.3	0.21		
	***di*-2**	0	0.0	0.0	100.0	100.0	1.00	0.23	
		1	0.0	0.0	100.0	100.0	1.00		
		2	0.0	0.0	100.0	100.0	1.00		
		4	0.0	0.0	100.0	99.8	1.00		
		8	0.0	0.1	100.0	99.2	1.01		
		15	0.2	0.3	41.1	97.4	0.42		
		30	0.7	1.1	32.1	90.7	0.35		
		60	3.0	3.7	18.0	72.2	0.25		
		90	6.7	7.1	5.1	54.4	0.09		
		120	10.8	10.7	0.5	40.7	0.01		
		180	19.2	18.1	6.0	24.7	0.24		

Table S8. WFI calculations of S_N1 model.

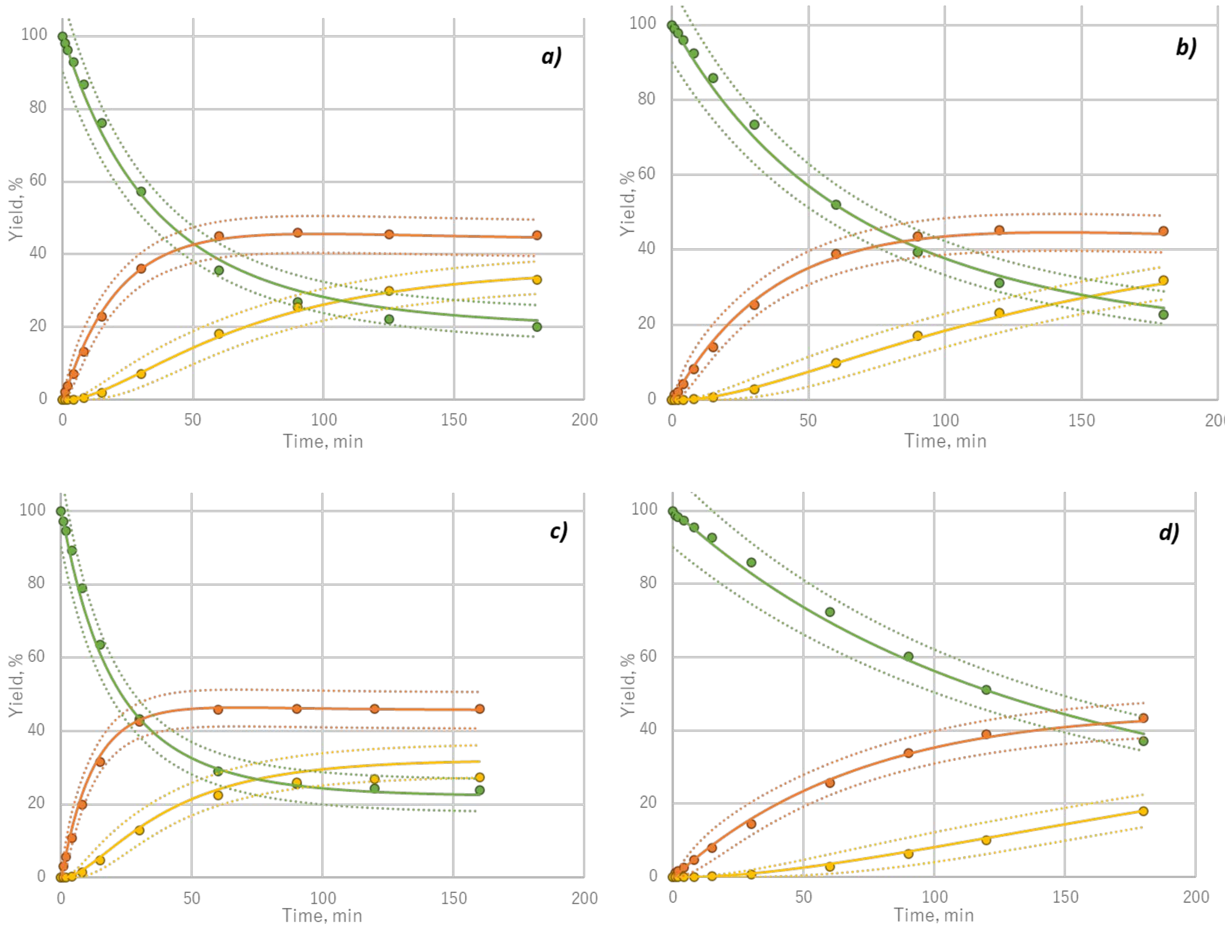

Figure S6. Overlay self-reproducibility plot of S_N1 mechanism model. Filled circles: experimental data; lines: simulation; dotted lines: weighted error range. Reaction conditions: DIPEA 1.5 eq, MeCN as solvent, *a*) 40°C, BnBr 1.2 eq; *b*) 30°C, BnBr 1.3 eq; *c*) 50°C, BnBr 1.1 eq; *d*) 20°C, BnBr 1.4 eq. Yields are HPLC conversion yield.

Borderline S_N mechanism.

No.	Elementary reaction
1	**1** + BnBr ⟶ **2** + HBr
2	HBr + ^{i}Pr$_2$NEt ⇄ ^{i}Pr$_2$NEt·HBr
3	**2** + BnBr ⟶ ***di*-2** + HBr
4	BnBr ⇄ Int
5	Int + **1** ⟶ **2** + HBr
6	Int + **2** ⟶ ***di*-2** + HBr

*Int = 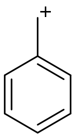

No.	*k* (fwd), L/mol.min	Ea (fwd), kJ/mol	Keq		Ea (back), kJ/mol
1	0.00105	102.4			
2	1000	60	1000	L/mol	60
3	0.0232	53.0			
4	0.0243	60	0.01	-	45
5	22.59	9.86			
6	7.44	5.87			

Table S9. Model parameters of borderline S_N model. Tref = 40℃ (for calculation of *k*)

Experiment	Series	Time, min	Y_e, %	Y_s, %	E_{abs}, %	E_{rel}, %	WFI(*n*)	WFI	Exp Ave
40℃, BnBr 1.2 eq	**1**	0	100.0	100.0	0.0	10.0	0.00	0.27	0.25
		1	98.0	98.0	0.0	10.0	0.00		
		2	96.1	95.9	0.2	10.0	0.02		
		4	92.9	91.9	1.0	10.0	0.10		
		8	86.7	84.6	2.5	10.0	0.25		
		15	76.2	73.7	3.4	10.1	0.34		
		30	57.2	56.6	1.1	10.3	0.11		
		60	35.7	38.0	6.1	12.0	0.51		
		90	26.8	29.4	8.9	14.8	0.60		
		125	22.2	24.5	9.3	17.8	0.52		
		182	20.0	21.1	5.6	20.9	0.27		
	**2**	0	0.0	0.0	100.0	100.0	1.00	0.14	
		1	2.2	1.9	12.4	84.1	0.15		
		2	3.8	4.0	3.9	70.4	0.06		
		4	7.2	7.8	8.5	51.2	0.17		
		8	13.3	14.5	8.1	31.1	0.26		
		15	22.9	23.6	2.6	18.5	0.14		
		30	36.4	35.4	2.9	12.6	0.23		
		60	45.2	44.0	2.9	11.1	0.26		
		90	46.2	45.8	0.8	10.9	0.07		
		125	45.7	46.0	0.8	10.9	0.07		
		182	45.6	45.6	0.1	10.9	0.01		
	***di*-2**	0	0.0	0.0	100.0	100.0	1.00	0.35	
		1	0.0	0.0	100.0	99.9	1.00		
		2	0.0	0.1	100.0	99.4	1.01		
		4	0.0	0.2	100.0	97.8	1.02		
		8	0.6	0.9	37.4	92.1	0.41		
		15	2.1	2.8	25.2	78.1	0.32		

		30	7.5	8.0	6.4	50.3	0.13		
		60	19.2	18.0	6.9	24.9	0.28		
		90	26.8	24.8	8.4	17.6	0.48		
		125	31.6	29.5	7.0	14.7	0.48		
		182	34.9	33.2	5.1	13.2	0.39		
30℃, BnBr 1.3 eq	**1**	0	100.0	100.0	0.0	10.0	0.00	0.13	0.17
		1	99.0	98.9	0.1	10.0	0.01		
		2	97.7	97.7	0.0	10.0	0.00		
		4	95.9	95.3	0.5	10.0	0.05		
		8	92.5	90.8	1.8	10.0	0.18		
		15	85.9	83.5	2.9	10.0	0.29		
		30	73.4	70.3	4.3	10.1	0.43		
		60	52.2	51.6	1.2	10.5	0.11		
		90	39.5	39.6	0.3	11.7	0.03		
		120	31.3	31.9	1.8	13.7	0.13		
		180	22.7	23.2	1.8	18.9	0.10		
	**2**	0	0.0	0.0	100.0	100.0	1.00	0.16	
		1	1.5	1.1	40.8	90.8	0.45		
		2	2.2	2.3	3.0	81.7	0.04		
		4	4.3	4.6	6.6	66.9	0.10		
		8	8.1	8.9	8.5	47.1	0.18		
		15	14.2	15.4	7.9	29.3	0.27		
		30	25.2	26.0	3.0	16.7	0.18		
		60	38.8	38.0	2.1	12.0	0.18		
		90	43.7	43.2	1.2	11.2	0.11		
		120	45.3	45.2	0.1	11.0	0.01		
		180	45.0	45.6	1.4	10.9	0.13		
	***di*-2**	0	0.0	0.0	100.0	100.0	1.00	0.22	
		1	0.0	0.0	100.0	100.0	1.00		
		2	0.0	0.0	100.0	99.8	1.00		
		4	0.0	0.1	100.0	99.2	1.01		
		8	0.2	0.3	44.5	97.1	0.46		
		15	0.7	1.1	36.4	90.8	0.40		
		30	2.7	3.6	24.7	72.5	0.34		
		60	9.9	10.4	5.2	41.8	0.12		
		90	17.1	17.2	0.3	26.2	0.01		
		120	23.3	22.9	1.7	19.1	0.09		
		180	31.8	31.2	1.9	14.0	0.13		

50℃, BnBr 1.1 eq	**1**	0	100.0	100.0	0.0	10.0	0.00	0.21	0.23
		1	97.2	96.6	0.6	10.0	0.06		
		2	94.6	93.0	1.7	10.0	0.16		
		4	89.2	86.5	3.1	10.0	0.31		
		8	78.9	75.5	4.5	10.0	0.45		
		15	63.5	61.1	3.8	10.2	0.38		
		30	43.3	43.2	0.2	11.2	0.02		
		60	29.1	29.5	1.3	14.7	0.09		
		90	25.4	25.0	1.8	17.4	0.10		
		120	24.3	23.3	4.5	18.7	0.24		
		160	23.9	22.5	6.3	19.5	0.32		
	**2**	0	0.0	0.0	100.0	100.0	1.00	0.18	
		1	3.1	3.4	7.6	74.1	0.10		
		2	5.8	6.8	14.8	55.6	0.27		
		4	10.9	12.8	14.5	35.1	0.41		
		8	20.1	22.1	9.0	19.8	0.45		
		15	31.8	32.6	2.5	13.5	0.18		
		30	42.7	42.2	1.3	11.3	0.12		
		60	46.0	45.8	0.3	10.9	0.03		
		90	46.3	46.0	0.8	10.9	0.07		
		120	46.2	45.9	0.8	10.9	0.08		
		160	46.3	45.8	1.1	10.9	0.10		
	***di*-2**	0	0.0	0.0	100.0	100.0	1.00	0.30	
		1	0.0	0.0	100.0	99.6	1.00		
		2	0.0	0.2	100.0	98.4	1.02		
		4	0.4	0.7	47.1	94.0	0.50		
		8	1.5	2.4	35.1	81.1	0.43		
		15	5.1	6.3	19.3	58.0	0.33		
		30	14.0	14.6	4.3	30.9	0.14		
		60	24.5	24.7	1.1	17.6	0.06		
		90	28.3	29.0	2.7	14.9	0.18		
		120	29.4	30.8	4.7	14.1	0.33		
		160	29.8	31.7	6.0	13.8	0.44		
20℃, BnBr 1.4 eq	**1**	0	100.0	100.0	0.0	10.0	0.00	0.17	0.24
		1	98.7	99.5	0.8	10.0	0.08		
		2	98.4	98.8	0.4	10.0	0.04		
		4	97.3	97.6	0.3	10.0	0.03		
		8	95.5	95.2	0.3	10.0	0.03		

	15	92.7	91.1	1.7	10.0	0.17	
	30	86.0	83.1	3.4	10.0	0.34	
	60	72.4	69.5	4.1	10.1	0.40	
	90	60.3	58.7	2.8	10.3	0.28	
	120	51.1	50.0	2.3	10.6	0.22	
	180	37.1	37.4	0.9	12.1	0.08	
**2**	0	0.0	0.0	100.0	100.0	1.00	0.27
	1	1.1	0.5	110.6	95.3	1.16	
	2	1.7	1.2	44.0	90.2	0.49	
	4	2.7	2.4	12.9	81.0	0.16	
	8	4.7	4.7	0.7	66.2	0.01	
	15	8.0	8.5	6.8	48.3	0.14	
	30	14.5	15.7	7.8	28.7	0.27	
	60	25.8	26.5	2.6	16.4	0.16	
	90	34.1	33.7	1.1	13.1	0.08	
	120	39.2	38.5	1.9	11.9	0.16	
	180	43.7	43.4	0.7	11.2	0.07	
***di*-2**	0	0.0	0.0	100.0	100.0	1.00	0.27
	1	0.0	0.0	100.0	100.0	1.00	
	2	0.0	0.0	100.0	100.0	1.00	
	4	0.0	0.0	100.0	99.8	1.00	
	8	0.0	0.1	100.0	99.2	1.01	
	15	0.2	0.3	45.2	97.2	0.47	
	30	0.7	1.2	37.2	89.9	0.41	
	60	3.0	4.0	24.4	70.3	0.35	
	90	6.7	7.6	12.2	52.0	0.24	
	120	10.8	11.5	6.5	38.4	0.17	
	180	19.2	19.2	0.1	23.2	0.01	

Table S10. WFI calculations of borderline model.
